# A multiparameter whole blood platform for assessment of anti-CMV immunity in allogeneic stem cell transplantation recipients

**DOI:** 10.3389/fimmu.2026.1872249

**Published:** 2026-09-11

**Authors:** Nina Imhof, Sebastian Wurster, Linda Heilig, Carolin Köchel, Hermann Einsele, Sabrina Kraus, Chris D. Lauruschkat

**Affiliations:** 1 Department of Internal Medicine II, University Hospital of Wuerzburg, Wuerzburg, Germany; 2 Department of Infectious Diseases, Infection Control and Employee Health, The University of Texas MD Anderson Cancer Center, Houston, TX, United States

**Keywords:** alloSCT, CMV reactivation, cytomegalovirus, immune monitoring, immune reconstitution, whole blood-based assay

## Abstract

Human cytomegalovirus (HCMV) infection remains a major challenge in allogeneic stem cell recipients. Current immune monitoring largely relies on IFN-γ-based readouts following *ex vivo* stimulation, which cannot fully capture the complex anti-HCMV immune defense. Therefore, we established a multiparameter HCMV-reactive whole blood (WB) assay that can be initiated at the bedside, requires minimal blood volume, preserves physiological cellular interactions, and enables integrated cellular and cytokine readouts within a closed-tube workflow. Using enhanced α-CD40 co-stimulation for robust detection of T-cell activation markers without widely used exocytosis inhibitors, our assay facilitates parallel flow cytometric quantification of activation-induced HCMV-reactive CD4^+^ and CD8^+^ T-cell responses and multiplexed detection of secreted cytokines. In parallel, our WB platform allows multimer-based detection of HCMV-specific CD8^+^ T cells and quantification of additional cell populations implicated in anti-HCMV protection, including Vδ1^+^ γδ T cells, “memory-like” natural killer (NK) cells, and HCMV-specific B cells. Across matched samples, readouts in WB and isolated peripheral blood mononuclear cells showed strong correlation. High intra-assay precision was achieved for key readouts across several immune cell compartments. Overall, our WB platform offers a simple, modular framework to advance post-transplant HCMV immune profiling while also being adaptable to other pathogens and immunological applications.

## Introduction

Letermovir prophylaxis through day 100 after allogeneic stem cell transplantation (alloSCT) has markedly reduced early clinically significant cytomegalovirus infection (csCMVi) and related complications in HCMV-seropositive alloSCT recipients (1). However, late-onset csCMVi after prophylaxis discontinuation remains a major clinical challenge (2). Assessment of HCMV-reactive cell-mediated immunity (CMI) has shown promise for risk stratification and may provide an avenue toward individualized management (3, 4). Current HCMV immune monitoring largely relies on IFN-γ-based assays following *ex vivo* antigen stimulation, with enzyme-linked immunospot assay (ELISpot), enzyme-linked immunosorbent assay (ELISA), and intracellular cytokine staining being the commonest methods (5).

Whole blood (WB)-based assays offer logistical advantages over those relying on isolation of peripheral blood mononuclear cells (PBMCs), because they avoid multiple time-consuming handling steps that may limit clinical applicability and introduce variability, commonly require smaller blood volumes, and preserve a more physiological stimulation environment (6). However, commercially available WB-based HCMV immunoassays such as QuantiFERON CMV showed varying performance across high-risk patient cohorts, with several studies reporting significant proportions of indeterminate results and overall modest capacity to predict csCMVi events (4, 7–12). In addition to technical constraints, this may reflect their narrow focus on IFN-γ^+^ type-1 HCMV-reactive T cells. While these cells are key correlates of HCMV control after alloSCT (5, 13), antiviral immunity is multidimensional and involves other cell populations that may provide complementary information for HCMV risk stratification and support individualized prophylactic and preemptive management strategies. Beyond conventional αβ T cells, γδ T cells may represent a relevant immune compartment for the assessment of antiviral immune reconstitution (3, 14). In particular, Vδ1^+^ γδ T cells have been associated with HCMV immunity. Their expansion has repeatedly been observed in HCMV-infected immunocompromised patients (15, 16) and delayed expansion of this cell population has been associated with an increased risk of csCMVi after alloSCT (3, 17). Consistent with these findings, reduced γδ T-cell frequencies at day 90 after alloSCT were recently associated with subsequent csCMVi, further supporting their potential relevance as an immune-monitoring marker (3). HCMV-expanded Vδ1^+^ γδ T cells exhibit functional properties of antiviral effector cells and may contribute to the control of infected cells through the production of cytokines such as IFN-γ and TNF-α as well as through cytotoxic mechanisms (18).

Natural killer (NK) cells are among the earliest lymphocyte populations to recover after alloSCT and may contribute to antiviral control before full restoration of adaptive immunity (19). Within the NK cell compartment, a subset termed “memory-like” NK cells have potent anti-HCMV activity. Following HCMV exposure, these cells expand and contribute to antiviral immunity through direct cytotoxicity, production of antiviral cytokines such as IFN-γ, and antibody-dependent cellular cytotoxicity (ADCC) (20–24). Since impaired NK cell reconstitution has been associated with HCMV-related complications after alloSCT, monitoring “memory-like” NK cells may provide additional complementary information on antiviral immune competence (3, 21).

Beyond cellular immunity, HCMV-specific B-cell responses and antibodies constitute an important component of antiviral immunity and have been associated with protection against clinically relevant HCMV infection as well as long-term viral control (25–27). Progressive B-cell reconstitution after alloSCT may support the development of protective humoral immunity, including neutralizing antibody responses, cytokine production, CD16-mediated recognition of virions, and ADCC (3, 28). Although humoral immunity has received comparatively little attention in HCMV immune monitoring after alloSCT because of delayed B-cell reconstitution, it may become increasingly relevant in the era of letermovir prophylaxis. Letermovir has shifted clinically significant HCMV reactivations toward later time points, frequently after prophylaxis discontinuation at day 100, when B-cell reconstitution is more advanced. Consequently, HCMV-specific memory B cells may provide complementary information for a more comprehensive assessment of HCMV-specific immunity in this setting (3).

Collectively, these findings suggest that incorporating additional immune compartments beyond conventional HCMV-reactive αβ T-cell measurements via IFN-γ may improve the characterization of HCMV-specific immunity. Whole blood-based platforms may facilitate such multiparametric immune profiling by enabling the simultaneous assessment of multiple cellular populations from a single sample while preserving the physiological cellular environment (3, 14–23, 25–28). Therefore, we herein establish a modular multiparameter WB assay with enhanced CD40 costimulation that eliminates the need for exocytosis inhibitors, enabling near-point-of-care handling and integrated analysis of multiple leukocyte lineages from a single sample. Specifically, our novel WB immune monitoring platform allows for combined analysis of HCMV-reactive T-cells via activation-induced markers or peptide multimers, multiplexed cytokine profiling, and quantification of “memory-like” NK cells, Vδ1^+^ γδ T cells, and HCMV-specific B cells within a same-day workflow.

## Materials and methods

### Informed consent

This study was approved by the Ethics Committee of the University of Würzburg, Germany (protocol code 17/19-sc). Written informed consent was obtained from all participants.

### Study population

Lithium heparin-anticoagulated peripheral blood (up to 18 mL) was collected at the University Hospital of Würzburg, Germany, between April 2025 and January 2026, three months to a maximum of two years after alloSCT.

**Figure 1:**
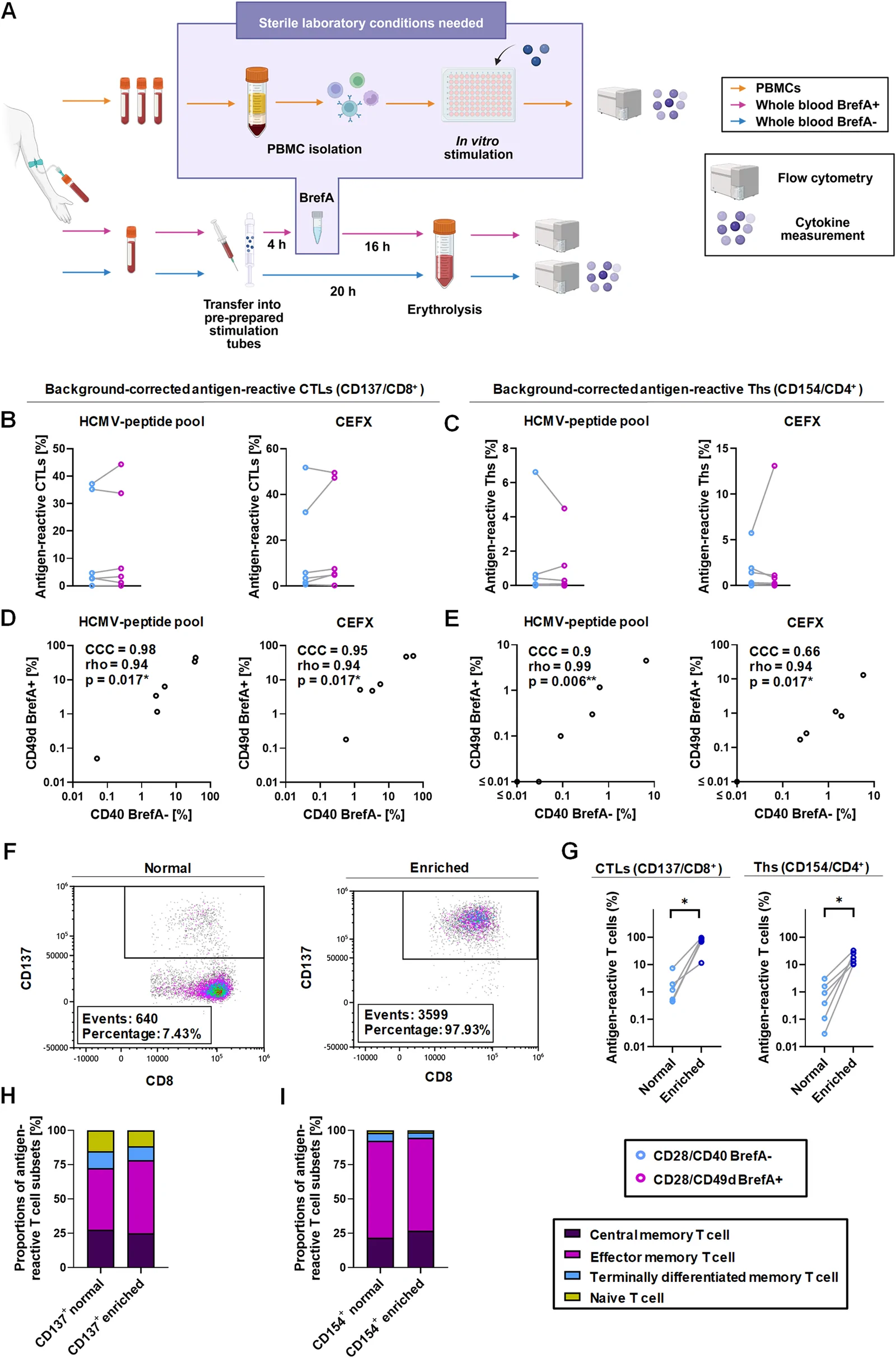
Replacement of Brefeldin A with α-CD40 maintains comparable frequencies of antigen-reactive T cells in the whole blood assay and enables magnetic bead enrichment for detailed subset analysis. **(A)** Schematic workflow illustrating the three stimulation conditions analyzed in this study. **(B–E)** Paired samples were costimulated either with α-CD28/α-CD40 without Brefeldin A (blue) or with α-CD28/α-CD49d with Brefeldin A (pink). **(B, C)** Frequencies of antigen-reactive cytotoxic T lymphocytes [CTLs; CD137^+^ of CD8^+^ cells, **(B)**] and T helper cells [Ths; CD154^+^ of CD4^+^ cells, **(C)**] following stimulation with the HCMV-peptide pool or CEFX. **(D, E)** Correlation between both conditions for HCMV-peptide pool and CEFX of CTL frequencies **(D)** and of Th frequencies **(E)**. **(F)** Representative flow cytometry plots showing CTL enrichment with (right) and without (left) antigen-specific T cell enrichment (ARTE). **(G)** Comparison of frequencies of HCMV-reactive CTLs (left) and T helper cells (right) enriched (dark blue) versus normal (light blue). **(H, I)** Proportions of memory subsets within CTLs **(H)** and Ths **(I)** with and without ARTE. Paired comparisons between samples were analyzed using the Wilcoxon signed-rank test **(B, C, G)**, correlations were assessed using Spearman’s rank correlation and concordance was assessed using Lin’s concordance correlation coefficient (CCC) **(D, E)**. *p < 0.05. **(B–E)** N = 6. HCMV-seropositive recipients analyzed between days 129 and 239 after alloSCT. **(G–I)** N = 6 HCMV-seropositive recipients analyzed between days 92 and 126 after alloSCT. alloSCT, allogeneic stem cell transplantation; ARTE, antigen-reactive T cell enrichment; CCC, Lin’s concordance correlation coefficient; CTLs, cytotoxic T lymphocytes; HCMV, human cytomegalovirus; MBCs, memory B cells; Ths, T helper cells.

**Figure 2:**
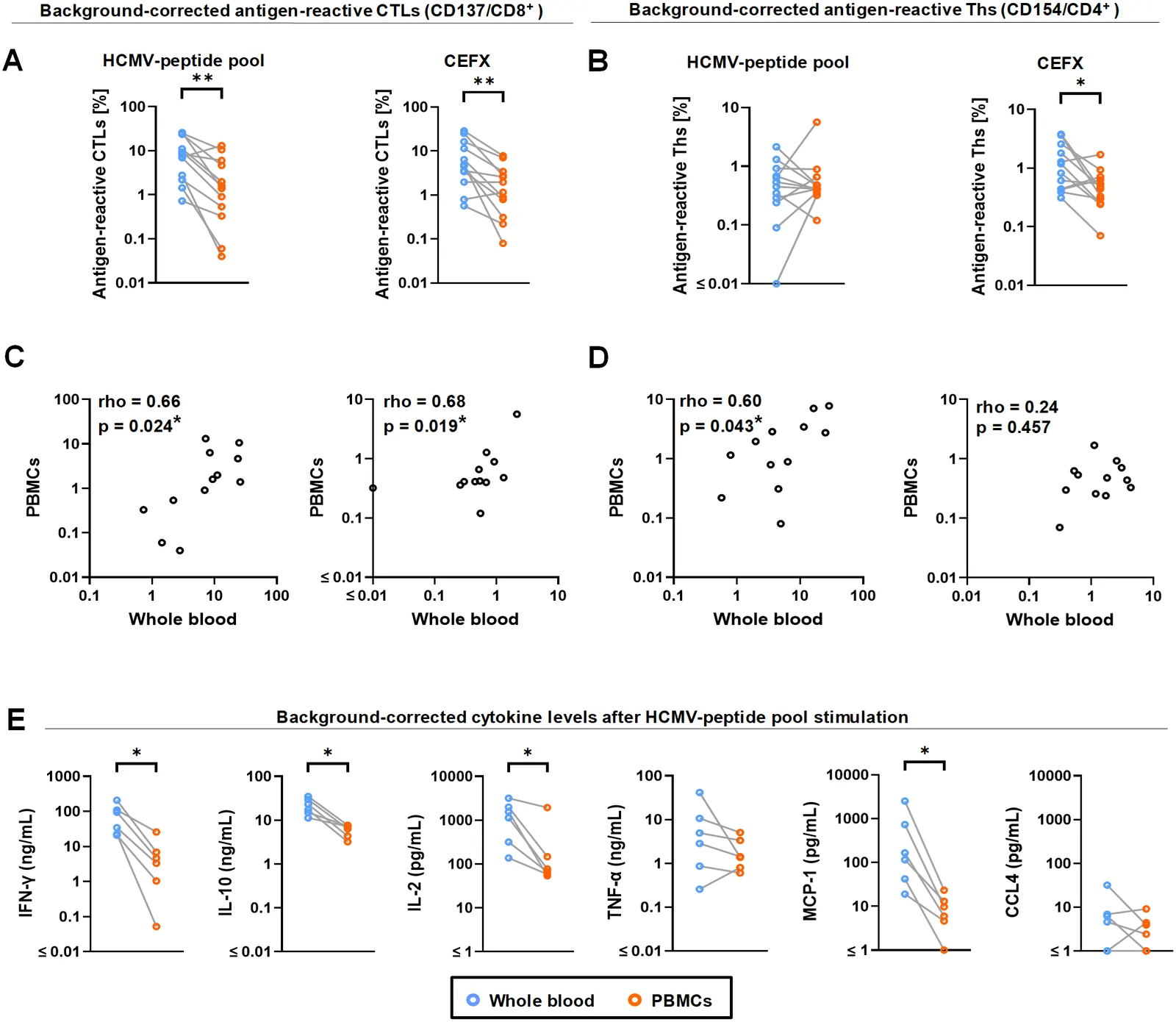
Whole blood stimulation detects higher frequencies of antigen-reactive T cells than PBMC stimulation, with strong correlation between methods and simultaneous measurement of diverse cytokines. **(A–E)** Analysis of paired whole blood (WB, blue) and PBMC (orange) samples both using α-CD28/α-CD40 co-stimulation. Comparison of frequencies of antigen-reactive cytotoxic T lymphocytes (CTLs; CD137^+^ among CD8^+^, **(A)**) and antigen-reactive T helper cells (Ths; CD154^+^ among CD4^+^, **(B)**) after stimulation with HCMV-peptide pool or CEFX. **(C, D)** Correlation of antigen-reactive CTL frequencies **(C)** and Th frequencies **(D)** between WB and PBMCs for HCMV-peptide pool and CEFX. **(E)** Plasma concentration of MCP-1, IL-2, IFN-γ, TNF-α, IL-10 and CCL4 after stimulation with HCMV-peptide pool for WB and PBMCs measured using a bead-based multiplex assay. Paired comparisons between samples were analyzed using the Wilcoxon signed-rank test **(A, B, E)**, and correlations were assessed using Spearman’s rank correlation **(C, D)**. *p < 0.05, **p < 0.01. **(A–E)** N = 12 **(A-D)** or n = 6 **(E)** HCMV-seropositive recipients analyzed between days 141 and 679 after alloSCT. alloSCT, allogeneic stem cell transplantation; CTLs, cytotoxic T lymphocytes; HCMV, human cytomegalovirus; IFN-γ, interferon gamma; IL, interleukin; MCP-1, monocyte chemoattractant protein-1; PBMCs, peripheral blood mononuclear cells; Ths, T helper cells; TNF-α, tumor necrosis factor alpha; WB, whole blood.

**Figure 3:**
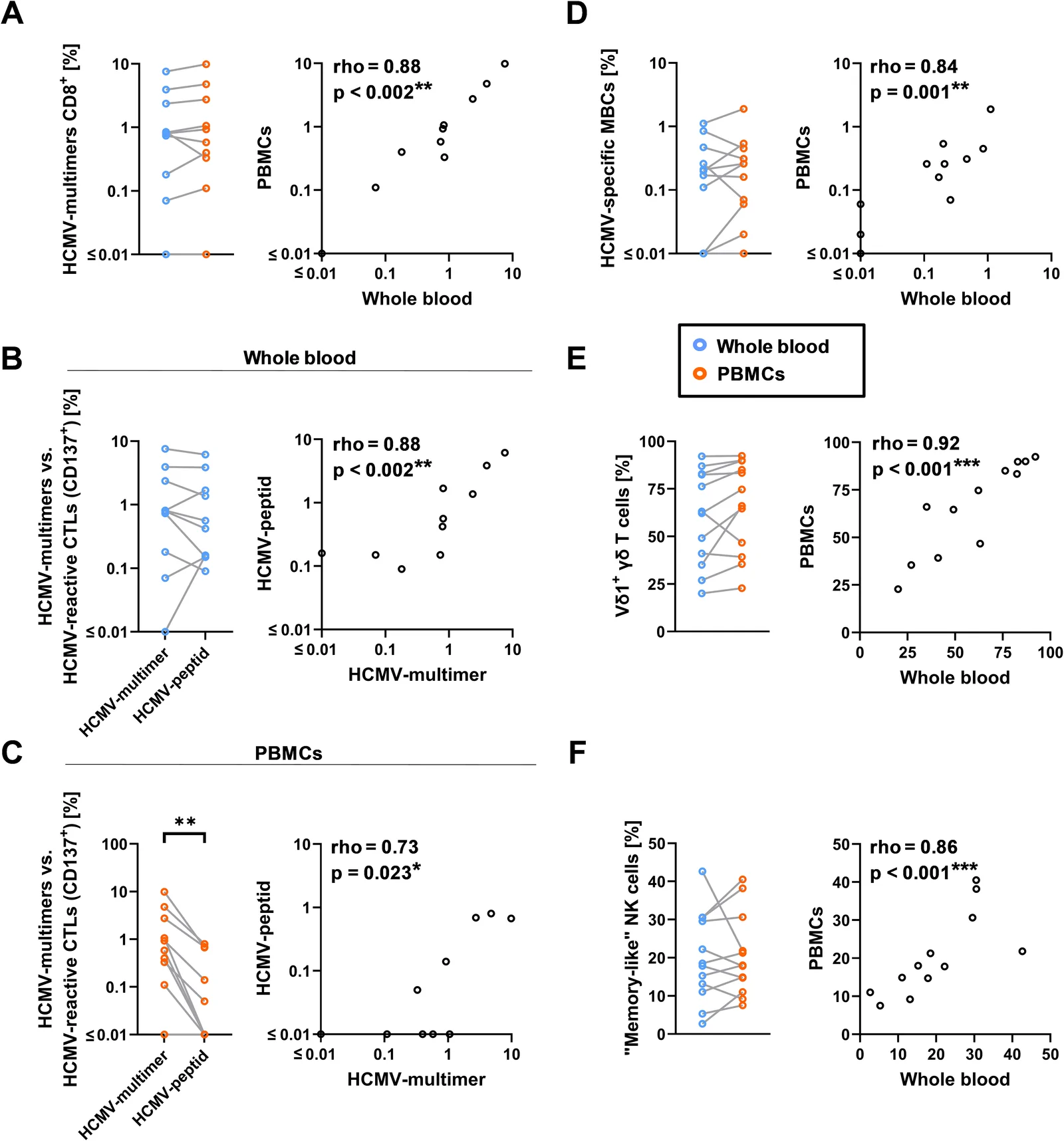
Reliable detection of HCMV-specific CTLs and non T-cell HCMV-protective immune populations in unstimulated whole blood. **(A)** Comparison of frequencies of HCMV-reactive CD8^+^ cytotoxic T lymphocytes (CTLs; multimer^+^ among CD8^+^) using HLA-matched HCMV-multimers in whole blood (WB, blue) and PBMCs (orange) (left) with corresponding correlation (right). **(B, C)** Comparison of frequencies of HCMV-specific CD8^+^ T cells identified either by HLA-matched multimers (multimer^+^ among CD8^+^ T cells) or by an activation-induced marker after peptide stimulation (CD137^+^ among CD8^+^ T cells) in WB **(B)** or PBMCs **(C)**, with the corresponding correlations shown in the right panels. **(D–F)** Comparison of HCMV-specific MBCs [among MBCs, **(D)**], Vδ1^+^ γδ T cells [among γδ T cells, **(E)**] and “memory-like” NK cells [among NK cells^dim^, **(F)**] frequencies (left) and correlations (right) between PBMC (orange) and WB (blue). **(A-F)** N = 10 **(A-C)** or N = 12 **(D-F)** HCMV-seropositive recipients analyzed between days 141 and 276 after alloSCT. **(A-F)** Paired comparisons between samples were analyzed using the Wilcoxon signed-rank test, and correlations were assessed using Spearman’s rank correlation. *p < 0.05, **p < 0.01, ***p < 0.001. alloSCT, allogeneic stem cell transplantation; AIM, activation-induced marker; CTLs, cytotoxic T lymphocytes; HCMV, human cytomegalovirus; MBCs, memory B cells; NK cells, natural killer cells; PBMCs, peripheral blood mononuclear cells; WB, whole blood.

**Figure 4:**
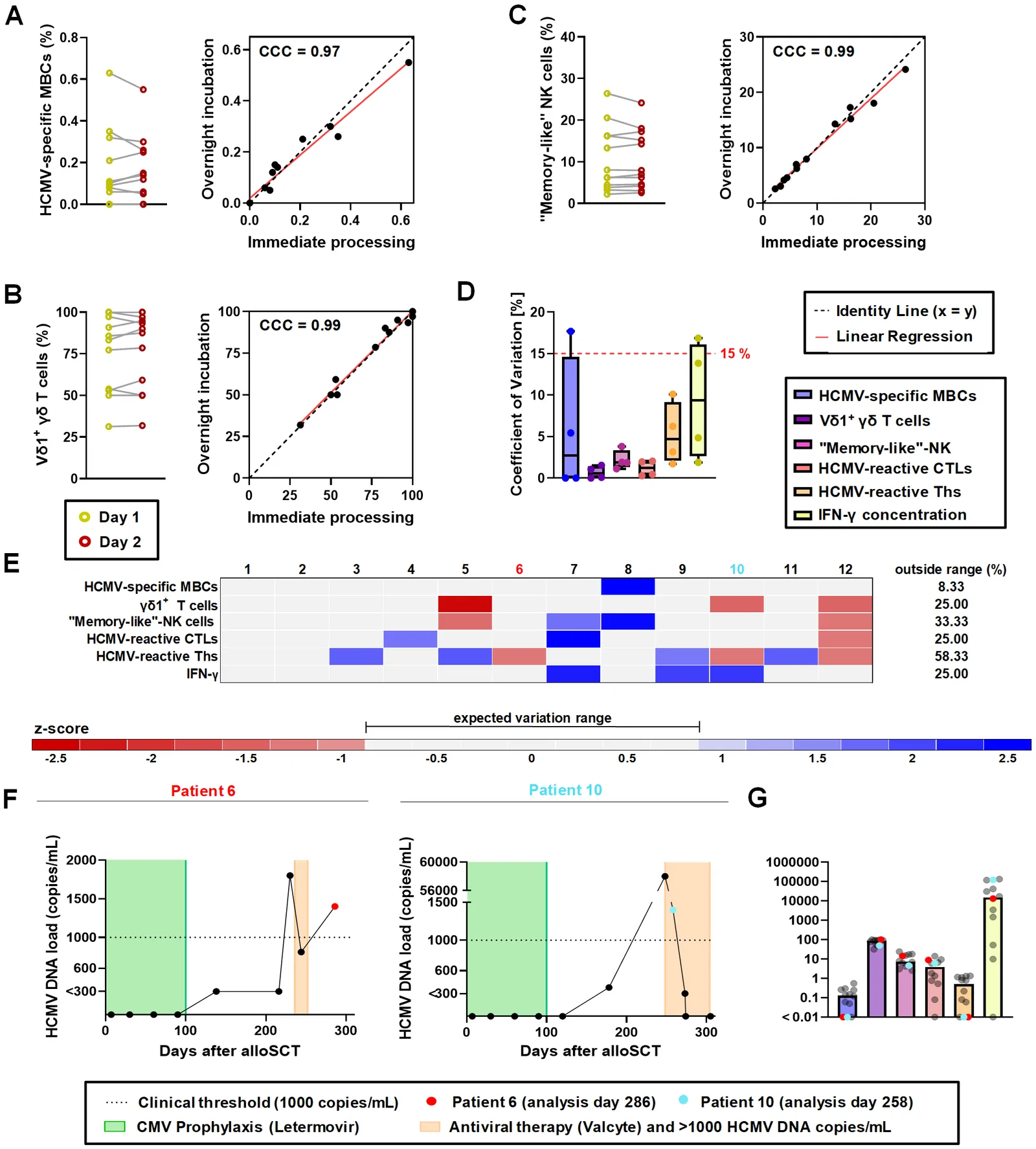
Simultaneous detection of diverse HCMV-protective immune populations by the whole blood assay with high intra-assay precision. **(A-C)** Comparison of frequencies of HCMV-specific memory B cells [MBCs; among MBCs, **(A)**], Vδ1^+^ T cells (among γδ T cells, **B**), and “memory-like” NK cells [among NK cells^dim^, **(C)**] (left) and corresponding scatter plots with identity line at 45° (dotted black) and linear regression line (red, right plot) measured in WB immediately (yellow) and after overnight incubation (red) after venipuncture. **(D)** Intra-assay precision (measured in duplicates) shown as coefficient of variation for HCMV-specific MBCs (blue), Vδ1^+^ γδ T cells (purple), “memory-like” NK cells (pink), as well as antigen-reactive cytotoxic T lymphocytes (CTLs; CD137^+^ among CD8^+^, red), antigen-reactive T helper cells (Ths; CD154^+^ among CD4^+^, orange), and IFN-γ levels (yellow) after HCMV-peptide pool stimulation. The acceptable FDA threshold of the coefficient of variation is indicated at 15% (red dotted line). **(E)** Heatmap of z-scored readouts (z-scored values calculated by subtracting the mean and dividing by the standard deviation) for all patients after overnight stimulation with defined outside range of ±1. **(F)** Longitudinal course of HCMV DNAemia for the two patients with csCMVi highlighted in the heatmap (patient 6, red; patient 10, blue). HCMV prophylaxis (green), antiviral treatment with HCMV DNAemia values exceeding 1,000 copies/mL (orange), and the clinical threshold of 1000 copies/mL (dotted line) are indicated. **(G)** Absolute values of all quantified immune parameters measured within the one-day workflow, with patient 6 (red dots) and patient 10 (blue dots) highlighted. **(A–C)** Concordance was assessed using Lin’s concordance (CCC). **(A–C, E)** N = 12; **(D)** n = 4. HCMV-seropositive individuals between day 182 and 656 after alloSCT were measured. alloSCT, allogeneic stem cell transplantation; CCC, Lin’s concordance correlation coefficient; csCMVi, clinically significant cytomegalovirus infection; CTLs, cytotoxic T lymphocytes; HCMV, human cytomegalovirus; IFN-γ, interferon gamma; MBCs, memory B cells; Ths, T helper cells; NK cells, natural killer cells; WB, whole blood.

Thirty-nine alloSCT recipients receiving letermovir prophylaxis until day 100 post-transplant were included in the study. Of these, samples from 27 patients were used for **Figures 1**–**3**; **Supplementary Figure 2**, whereas samples from 12 patients were analyzed for the simultaneous detection of diverse HCMV-protective immune cell populations within a same-day workflow (**Figure 4**). Pharmacological immunosuppressive therapy at the time of immune monitoring was documented as a potential confounding factors. However, only a minority of patient were receiving immunosuppressive therapy at the time of sampling (**Table 1**).

**Table 1:** Characteristics of allogeneic stem cell transplant recipients included for **Figures 1**-**3** and **Supplementary Figure 2** and **Figure 4**.

Variables	Figures 1-3, Supplementary Figure S2 N = 27	Figure 4 N = 12
Age, years, median	64	58
Sex, N (%)		
Male	14 (52)	6 (50)
Female	13 (48)	6 (50)
Underlying disease, N (%)		
Acute leukemia	18 (67)	8 (67)
Chronic leukemia	1 (4)	0 (0)
Lymphoma	1 (4)	1 (8)
Multiple myeloma	0 (0)	0 (0)
Others	7 (26)	3 (25)
HLA matching, N (%)		
Matched related	1 (4)	0 (0)
Matched unrelated	21 (78)	10 (83)
Mismatch	5 (19)	2 (17)
Haploidentical	0 (0)	0 (0)
Stem cell source, N (%)		
Bone marrow	0 (0)	1 (8)
PBSC	27 (100)	11 (92)
Conditioning intensity, N (%)		
Reduced intensity (RIC)	24 (89)	11 (92)
Myeloablative (MAC)	3 (11)	1 (8)
Antithymocyte globulin, N (%)		
No	2 (7)	0 (0)
Yes	25 (93)	12 (100)
HCT-CI, N (%)		
0-2	16 (59)	8 (67)
3-4	8 (30)	4 (33)
≥5	3 (11)	0 (0)
csCMVi, N (%)		
Previous csCMVi	6 (22)	3 (25)
Ongoing csCMVi (at the time of sampling)	0 (0)	2 (17)
No	21 (78)	9 (75)
aGvHD, N (%)		
0-1	13 (48)	6 (50)
2-4	14 (52)	6 (50)
cGvHD, N (%)		
No	16 (59)	7 (58)
Yes	11 (41)	5 (42)
HCMV serostatus (R/D), N (%)		
+/+	18 (67)	8 (67)
-/-	3 (11)	0 (0)
+/-	6 (22)	4 (33)
Steroid use*, N (%)		
None	15 (56)	8 (67)
0 to ≤1 mg/kg/d	4 (15)	1 (8)
2 mg/kg/d	8 (30)	3 (25)
Immunosuppressive therapy at the time of sampling, N (%)		
No	20 (74)	10 (83)
Yes	7 (26)	2 (17)

HCMV, human cytomegalovirus; csCMVi, clinically significant cytomegalovirus infection; HLA, human leukocyte antigen; PBSC, peripheral blood stem cells; HCT-CI, Hematopoietic Cell Transplantation Comorbidity Index; GvHD, graft-versus-host disease; R, recipient; D, donor; S2, **Supplementary Figure 2**.

*Divided into 0, 0 to ≤1, or 2 mg/kg/day based on maximum steroid dose during the study period.

### WB-based stimulation assay

WB stimulation tubes were prepared and stored following our published protocols (6, 29, 30). The following stimulation conditions were assessed: unstimulated negative control, HCMV-peptide pool CD4^+^ & CD8^+^ (2 µg/mL; Mabtech, Inc. Nacka Strand, Sweden), CEFX-1 (1µg/mL; JPT Peptide Technologies GmbH, Berlin, Germany), and HCMV-derived peptide stimulation (0.1 µg/mL; JPT) according to the patient’s human leukocyte antigen (HLA) type. Costimulatory antibodies α-CD28+α-CD40 or α-CD28+α-CD49d (1 µg/mL each, Miltenyi Biotec, Bergisch Gladbach, Germany) were added.

0.45 mL lithium heparin-anticoagulated peripheral blood was aseptically transferred into pre-prepared stimulation tubes using a sterile, graded 1-mL syringe. The α-CD28+α-CD49d condition was further supplemented with a final brefeldin A (BrefA) concentration of 10 µg/mL (Thermo Scientific, Waltham, MA, USA) after 4 h of incubation, as described previously (6). All samples were incubated for a total of 20-22 h at 37 °C. Thereafter, tubes were centrifuged at 300×*g* for 10 minutes, and the plasma was stored at -80 °C until further use.

### Erythrocyte lysis and activation-induced marker (AIM)-based flow cytometry for antigen-reactive T cells

After incubation, erythrocytes were lysed (as described in detail in the **Supplementary Methods** section 2) with buffer volumes adjusted according to the initial blood volume. Cells were stained extracellularly with antibodies listed in **Supplementary Table 1** for 30 min. After two washing steps, cells were fixed and permeabilized using the Inside Staining Kit (Miltenyi Biotec) according to the manufacturer’s instructions. Intracellular staining was performed using CD137-PE and CD154-APC (both Miltenyi Biotec) for 10 min at room temperature in the dark. Following two additional washing steps, cells were analyzed by flow cytometry. Representative gating strategies for the identification of antigen-reactive T cells are shown in **Supplementary Figure 3** for WB and **Supplementary Figure 4** for PBMCs. Background correction was performed by subtracting the antigen-reactive frequencies measured in the corresponding unstimulated controls from those obtained under stimulated conditions for each patient and each respective condition.

To assess the detectability of different activation markers in our whole blood AIM assay without BrefA and with α-CD28/α-CD40 co-stimulation, CD25, HLA-DR, and CD134 were added to the staining panel. Experimental details are provided in the **Supplementary Methods** (section 3) and the corresponding gating strategy (**Supplementary Figure 5**).

### PBMC-based assay

PBMC isolation and stimulation were performed as described before (21). Costimulatory factors and antigen stimuli were aligned with the WB-based assay.

### Cytokine quantification

Cytokine concentrations in plasma or PBMC supernatant were quantified using LEGENDplex (BioLegend; MCP-1, IL-2, IFN-γ, TNF-α, CCL4, and IL-10) according to the manufacturer’s instructions (6).

### Analysis of unstimulated immune cell subsets: γδ T cells, NK cells, B cells, and HLA-reactive multimer detection

In parallel to the antigen-stimulated assay, 3 mL blood was processed immediately after collection for baseline immune phenotyping. Erythrocyte lysis was performed as described above, with buffer volumes adjusted according to blood volume. Cells were suspended in Roswell Park Memorial Institute (RPMI) medium + 5% autologous human serum and counted using an automated cell counter (Countess 3; Invitrogen Corporation, Carlsbad, CA, USA).

For staining of γδ T cells and NK cells, 3×10^5^ PBMCs or leukocytes from the WB cell pellet were used and stained as described in **Supplementary Methods** (section 4). For multimer-based detection of antigen-reactive CD8^+^ T cells, 5×10^5^ PBMCs or WB-derived leukocytes were stained according to the patient’s HLA type using MACSimer multimers (Miltenyi Biotec, 10 mmol/L) for HLA*A01:01, HLA*A02:01, or HLA*B07:02 loaded with the respective HCMV derived peptides [CMV UL44 (VTEHDTLLY) HLA*A01:01, CMVpp65 (NLVPMVATV) HLA*A02:01, CMVpp65 (RPHERNGFTVL) HLA*B07:02; all Miltenyi Biotec]. The corresponding unloaded HLA*A01:01, HLA*A02:01, or HLA*B07:02 multimers served as negative controls. A detailed description of the procedure is provided in the **Supplementary Methods** (section 5).

The gating approach applied for the identification of γδ T cells and NK cells is illustrated in **Supplementary Figure 6** for WB and **Supplementary Figure 7** for PBMCs. The corresponding gating strategies for multimer-based analyses are provided in **Supplementary Figure 8** for WB and **Supplementary Figure 9** for PBMCs.

HCMV-specific memory B cells (MBCs) were identified by staining 5×10^6^ PBMCs or WB-derived leukocytes with a panel of biotinylated HCMV glycoprotein probes, consisting of trimer (gH/gL/gO complex, The Native Antigen Company, Kidlington, UK), pentamer (gH, gL, UL128, UL130, UL131A complex, The Native Antigen Company), and glycoprotein B (gB, Sino Biological, Beijing, China), each conjugated with streptavidin fluorophores, as described previously (3). Detailed information on the probe generation and staining procedure is provided in the **Supplementary Methods** (section 6). Representative gating strategies for the detection of HCMV-specific MBCs are shown for WB and PBMCs in **Supplementary Figures 10** and **11**, respectively.

To assess whether analyses can be performed reliably after overnight incubation for optimal alignment with antigen-stimulated assays, 0.45 mL of WB was added to antigen-free stimulation tubes containing 50 µL of RPMI medium for analysis of γδ T cells and NK cells. For HCMV-specific B cells, 1.8 mL of WB was added to tubes containing 200 µL of RPMI medium. Samples were incubated at 37 °C overnight and subsequently processed for staining as described in the **Supplementary Methods** (section 7-8).

### HCMV-reactive T-cell enrichment

HCMV antigen-reactive T cell enrichment (ARTE) was performed according to published protocols (31–34) and **Supplementary Methods** (section 9). Cells were then further stained with additional antibodies for downstream analyses. The gating strategy used for this panel is provided in **Supplementary Figure 12**.

### Flow cytometry

Stained cells were analyzed on a CytoFlex flow cytometer using CytExpert v.2.4 software (Beckman Coulter, Brea, CA, USA). Data analysis was performed with Kaluza (version 2.2.1, Beckman Coulter).

### Supplementary methods

Detailed protocols for all assays, antibodies and other key reagents, and gating strategies are provided in **Supplementary Methods**.

### Statistical analysis and software

Significance was tested using the Mann-Whitney U test or Wilcoxon matched-pairs signed rank test, as appropriate. Correlation and concordance were assessed using Spearman’s rank correlation and Lin´s concordance correlation coefficients. Data were compiled, visualized, and analyzed using Excel 365 (Microsoft, Redmond, WA, USA), Prism v.10.4.2 (GraphPad Software, Boston, MA, USA), and R v.4.5.1. (R Core Team, 2025).

## Results

### Optimized WB assay platform for parallel AIM and cytokine-based quantification of antigen-specific T-cell responses

Exocytosis inhibitors like BrefA are commonly used for optimal AIM-based detection of HCMV-reactive cytotoxic T lymphocytes (CTLs) and T helper cells (Ths), especially for CD154-based detection of antigen-specific Th cells (6, 21). However, BrefA prevents simultaneous cytokine measurement and must be added shortly after stimulation, limiting clinical practicability. In PBMCs, α-CD40 combined with α-CD28 was shown to prevent CD154 internalization without BrefA (35). To enable a multiparameter HCMV-WB assay near the point of care, we recapitulated this strategy in our WB platform and compared it to our previous protocol that used a combination of α-CD28, α-CD49d, and BrefA (**Figure 1A**).

For both Th cells and CTLs, background-corrected antigen-reactive T-cell frequencies were comparable between both protocols for both the HCMV-peptide pool (median, CD137^+^ CTLs: 3.76 vs 4.87; CD154^+^ Th cells: 0.27 vs 0.2) and CEFX, a broad viral peptide pool commonly used to assess global T-cell responsiveness (n=6; median, CD137^+^ CTLs: 4.52 vs 6.27; CD154^+^ Th cells: 0.88 vs 0.55) (**Figures 1B, C**). Positive response thresholds were defined as the median plus two standard deviations of HCMV-seronegative controls, according to Dan et al. (36), resulting in cut-off values of 0.02% for HCMV-specific Ths responses and 0.31% for HCMV-specific CTL responses. The two protocols showed strong correlations across all T-cell populations and stimuli (rho = 0.94–0.99; p = 0.006–0.017) and high concordance, including for responses stimulated with the HCMV-peptide pools (CCC = 0.90–0.98). Lower concordance was observed only for CEFX-reactive Th-cell responses (CCC = 0.66), which are not used for the prediction of csCMVi (**Figures 1D, E**). In addition, there was a trend toward higher unstimulated background frequencies for CTLs in the presence of BrefA (median, 0.07 vs. 0.225; p = 0.063), whereas no significant difference was observed for Th cells (**Supplementary Table 2**). Collectively, these findings indicate that BrefA can be omitted without substantially affecting assay performance for HCMV-peptide pool-stimulated responses.

While many AIM assays identify antigen-reactive T cells using especially CD154 (Ths) via single activation markers (31, 32, 37), combinatorial AIM marker strategies are increasingly being used. Therefore, we assessed whether additional activation markers and their combinations could be detected in our whole blood assay using BrefA-free WB assay with α-CD28/α-CD40 co-stimulation. Following HCMV peptide pool stimulation, all additionally investigated activation markers, CD69, CD134, CD25, and HLA-DR, were detectable both individually and in combination with CD137^+^ and CD154^+^ (n=6, **Supplementary Figure 2**).

Antigen-specific T-cells are often rare, particularly early after transplantation, potentially resulting in unreliable phenotypic characterization of rare populations. Therefore, we adapted the ARTE antigen-specific T cell enrichment workflow from PBMCs to WB, evaluating enrichment of CD137^+^ HCMV-reactive CTLs and CD154^+^ Ths based on AIM expression. Enrichment was performed successfully on both populations (n=6), with median frequencies increasing from 0.88 to 77.63 for HCMV-reactive CTLs (p=0.031) and from 0.72 to 17.5 (p=0.031) for HCMV-reactive Th cells, respectively (**Figures 1F, G**), indicating strong and significant enrichment. Notably, memory phenotypes of HCMV-specific Th cells and CTLs were highly consistent in baseline and post-enrichment measurements (**Figures 1H, I**).

Since PBMC-based assays are commonly used as reference for antigen-reactive T-cell measurements, we also compared the performance of the BrefA-free WB assay with α-CD28/α-CD40 co-stimulation against a corresponding PBMC-based protocol using paired specimens (n=12) from the same patients. Here, the WB assay detected significantly higher antigen-reactive CTL frequencies for both HCMV-peptide pool (median, 7.81 vs 1.5, p=0.007) and CEFX (median, 4.74 vs 1.56; p=0.002) as well as higher CEFX-reactive Th frequencies (median, 1.15 vs 0.46, p=0.012) (**Figures 2A, B**). Additionally, a strong positive correlation between WB- and PBMC-based readouts was found following HCMV-peptide pool stimulation (rho=0.60-0.66, p=0.024-0.043) (**Figures 2C, D**). Importantly, the higher antigen-reactive T-cell frequencies detected in the WB assay were not accompanied by significantly increased background activation (**Supplementary Table 2**).

Because BrefA blocks cytokine secretion, its omission enables simultaneous AIM and cytokine quantification to complement immune-guided risk stratification. We therefore performed multiplex cytokine profiling in paired WB and PBMC samples from HCMV-seropositive patients (n=6), measuring MCP-1, IL-2, IFN-γ, TNF-α, CCL4, and IL-10. All analytes were readily detectable following HCMV-peptide pool stimulation. Compared with PBMCs, WB showed significantly higher concentrations of MCP-1, IL-2, IFN-γ, and IL-10, whereas TNF-α and CCL4 showed no significant differences between the two platforms (e.g., IFN-γ: 64.3 vs. 4.0 ng/mL, p=0.031) (**Figure 2E**). To preclude false-positive detection, we also tested cytokine levels in HCMV-seronegative patients after HCMV-peptide pool stimulation, which remained within the range observed in unstimulated samples from HCMV-seropositive patients (**Supplementary Figure 1A**).

Overall, omission of BrefA enabled combined assessment of antigen-reactive T-cell frequencies and multiplexed cytokine quantification, yielding higher signals for most readouts in WB, strong correlation with PBMC-based readouts, and a workflow better suited for clinical applications.

### Quantification of additional HCMV-protective immune cell populations without prior stimulation and alignment of workflows for multifaceted immune phenotyping

To complement antigen-stimulated workflows, we additionally assessed the feasibility of detecting HCMV-specific CTLs without prior stimulation in whole blood using HLA-restricted peptide multimers. Therefore, blood from alloSCT recipients (n=10) carrying HLA*A01:01, HLA*A02:01, or HLA*B07:02 was stained with the corresponding multimers loaded with the HCMV-derived peptides. HCMV-specific CTLs (median, 0.64 multimer^+^ CD8^+^ cells) were detected exclusively in HCMV-seropositive recipients (**Supplementary Figure 1B**). In paired PBMC and WB samples, similar HCMV-reactive multimer^+^ CTL frequencies were detectable in both WB and PBMC-based assays and showed showed a strong positive correlation between platforms (rho=0.88, p<0.002) (**Figure 3A**) Comparison of multimer^+^ CTL frequencies with AIM frequencies following stimulation with the corresponding HCMV peptide revealed robust correlations in WB (rho=0.88, p<0.002; **Figure 3B**) and PBMCs (rho=0.73, p<0.023; **Figure 3C**).

HCMV-reactive T cells are central to antiviral control, yet protective immunity after alloSCT is multidimensional. Specifically, “memory-like” NK cells, Vδ1^+^ γδ T cells, and humoral responses showed associations with csCMVi protection (3, 5, 13, 21). We therefore assessed whether these immune compartments can be reliably quantified from WB (n=12). First, we evaluated HCMV-specific MBC detection in WB using our glycoprotein probe panel previously validated in PBMCs, which targets the major humoral HCMV antigens, including trimer (gH/gL/gO complex), pentamer (gH/gL/UL128/UL130/UL131A complex), and glycoprotein B (gB). In paired samples, HCMV-specific MBC frequencies were detectable in both WB and PBMCs and showed a high correlation between platforms (rho=0.86, p=0.001) (**Figure 3D**). No detectable signals were found in seronegative controls (**Supplementary Figure 1C**). Similarly, comparable median frequencies and strong positive correlation in WB and PBMCs were found for Vδ1^+^ γδ T cells (rho=0.92, p<0.001) and “memory-like” NK cells (rho=0.86, p<0.001) (**Figures 3E, F**).

To align workflows for a multifaceted WB platform amenable to both antigen-stimulated T-cell detection and quantification of additional cellular players, we tested whether HCMV-specific MBCs, Vδ1^+^ γδ T cells, and “memory-like” NK cells could be measured reliably from an unstimulated tube after overnight incubation in parallel with the AIM assay (n = 12). All immune cell subsets, including HCMV-specific MBCs (CCC = 0.97), Vδ1^+^ γδ T cells (CCC = 0.99), and “memory-like” NK cells (CCC = 0.99), showed near-optimal concordance between immediate quantification and measurement after 22 h of incubation, indicating that alignment of workflows is feasible (**Figures 4A–C**).

Next, we evaluated intra-assay precision for key parameters using the aligned overnight workflow. In four independent replicate measurements (n=4), median coefficients of variation were below 15% for all parameters, and the maximum CV remained below 20% (HCMV-specific MBCs: 2.72; Vδ1^+^ γδ T cells: 0.577; “memory-like”-NK cells: 1.88; HCMV-reactive CTLs: 1.2; HCMV-reactive Ths: 4.7 and IFN-γ: 9.35) (**Figure 4D**). Thus, the majority of measurements fell well within the FDA-defined 15% threshold for bioanalytical assays (38). Lastly, using the overnight-incubation workflow, we visualized multiple immune readouts per patient as z-scores (calculated as the deviation from the cohort mean divided by the standard deviation (39)). The resulting heatmap illustrates the feasibility of comprehensive, same-day immune cell profiling across multiple functional and cellular parameters in our platform in 12 alloSCT recipients (**Figure 4E**). To further contextualize the immune profiles visualized in the heatmap, we retrospectively evaluated the corresponding clinical parameters of patients displaying distinct immune signatures. Interestingly, the four patients with the most pronounced reduction across immune readouts (patients 5, 6, 10, and 12) had experienced or were experiencing virus-related events at the time of measurement. Notably, patients 6 and 10 developed csCMVi. To further characterize these cases, we analyzed the clinical course and HCMV-related parameters of both patients in detail. Both patients had received letermovir prophylaxis until day 100 after alloSCT and subsequently initiated valganciclovir treatment after detection of HCMV DNAemia exceeding 1000 copies/mL. Following valganciclovir treatment, patient 6 initially showed a transient decrease of HCMV DNAemia below 1000 copies/mL; however, at the time of immune profiling, HCMV DNA levels had increased again to >1,000 copies/mL. Due to referral to another center, no further clinical follow-up data were available for this patient. In contrast, patient 10 achieved control of HCMV replication while remaining on antiviral therapy (**Figure 4F**). Analysis of the absolute cell numbers revealed that neither alloSCT recipient with csCMVi had detectable HCMV-specific memory B cells or HCMV-reactive T helper cells (**Figure 4G**).

Altogether, these findings demonstrate that our WB platform enables a combined workflow for simultaneous, multiparameter assessment of HCMV-specific T and B cells, Vδ1^+^ γδ T cells, and “memory-like” NK cells with high precision and strong correlation with conventional, PBMC-based assays.

## Discussion

HCMV-CMI testing is an established approach for HCMV risk stratification after alloSCT, with prospective ELISpot data supporting its clinical utility and more recent letermovir-era studies confirming a close link between cellular immune reconstitution, HCMV control, and clinically significant infection (13, 21, 40, 41). Thus, HCMV-CMI may provide a framework to individualize the duration of antiviral prophylaxis (40, 41), as a CMI-guided strategy for individualized letermovir duration was shown to result in a lower incidence of late-onset csCMVi compared with a fixed 100-day course.

However, currently available HCMV-CMI approaches may have limited predictive performance for csCMVi in the letermovir era (8, 11). Specifically, in one of the largest HCMV-CMI studies in alloSCT recipients receiving letermovir prophylaxis, Zamora et al. analyzed 120 patients and found that a purely IFN-γ-based assay alone had a poor predictive performance for late-onset csCMVi (4). Although predictive performance improved when HCMV-CMI was combined with clinical variables such as early HCMV infection (4), this study underscored the need for more nuanced and multidimensional HCMV-CMI assays. Specifically, emerging data support a contribution of “memory-like” NK cells, Vδ1^+^ γδ T cells, and humoral immunity to HCMV control, potentially in synergy with conventional T-cell responses (3, 5, 13, 21). Higher pre-reactivation Vδ1^+^ γδ T-cell frequencies and stronger day-90 γδ T-cell reconstitution are associated with improved HCMV control, whereas low “memory-like” NK-cell numbers correlate with more severe HCMV manifestations (3, 21). The role of humoral immunity is also increasingly recognized, although its integration into combined immune-monitoring strategies remains to be studied further (5, 28).

Therefore, we herein developed a multiparameter WB assay that extends current IFN-γ-based CMV immune-monitoring by enabling parallel assessment of soluble and cellular immune readouts in a single platform. The assay captures HCMV-reactive Th cells and CTLs as well as immune populations that have been implicated in HCMV protection, including “memory-like” NK cells, Vδ1^+^ γδ T cells, and HCMV-specific B cells, along with antigen-responsive cytokine profiling. The cytokines quantified in our panel (MCP-1, IL-10, IL-2, IFN-γ, TNF-α, and CCL4) represent complementary immune mediators relevant to anti-HCMV responses (42, 43), and the panel could be expanded further (29).

Separate assessment of HCMV-specific Th cells, distinct from CTL-focused approaches, is especially relevant, given evidence that CD4^+^ T-cell immunity remains a key determinant of protective anti-HCMV responses after alloSCT (13, 44). Recovery of the CD4^+^ T-cell compartment represents an important indicator of immune reconstitution after alloSCT, with early global and HCMV-specific CD4^+^ T-cell recovery being consistently associated with reduced HCMV viremia and disease (45–48). Beyond serving as a marker of immune reconstitution, HCMV-specific CD4^+^ T cells are pivotal for antiviral control by supporting CD8^+^ T-cell expansion and maintenance, antiviral cytokine production, B-cell responses, viral clearance, and the establishment of durable antiviral memory (13, 49, 50). Along these lines, although this was not a primary aim of our study and the sample size was very limited, our WB assay revealed reduced frequencies of HCMV-specific CD4^+^ T-cells in both patients who developed csCMVi. Together, these findings further support monitoring HCMV-specific CD4^+^ T-cell immunity as a clinically relevant marker for predicting the risk of csCMVi after alloSCT.

Furthermore, we demonstrated the feasibility of AIM-based T-cell enrichment from WB samples to improve detection of low-frequency antigen-reactive T cells and enable more reliable phenotypic and functional characterization of rare populations such as multifunctional HCMV-reactive T cells (41), which could potentially inform personalized HCMV risk stratification and guide post-transplant monitoring, although the clinical value of these subpopulations requires further investigation.

Although HCMV-specific T-cell detection via IFN-γ remains an important marker of HCMV-specific T-cell immunity, increasing evidence indicates that antiviral immune competence after alloSCT is multidimensional and cannot be fully captured by a single functional readout (5, 8, 9). Combining cytokine-based functional readouts with additional cellular parameters may therefore provide complementary information on antiviral immune competence. In particular, Vδ1^+^ γδ T cells and “memory-like” NK cells represent promising candidates for expanded immune monitoring due to their association with HCMV control and clinically significant HCMV events (3, 15, 16, 19, 21). NK cells may contribute to early antiviral protection through enhanced HCMV-specific responsiveness and their rapid reconstitution after alloSCT (3, 19–21), whereas Vδ1^+^ γδ T cells provide rapid, MHC-independent recognition of infected cells and may compensate for delayed conventional T-cell recovery after transplantation (3, 15, 16, 18, 51).

Furthermore, polyfunctional HCMV-specific T-cell responses characterized by the coordinated production of cytokines such as IFN-γ, TNF-α, and IL-2 have been associated with protective antiviral immunity (52). These cytokines are also characteristic of functional CD4^+^ Th1 responses (53), which, as discussed above, are key contributors to effective HCMV control after alloSCT (13). Although our assay does not allow direct assessment of single-cell T-cell polyfunctionality, simultaneous measurement of these functionally relevant cytokines in the stimulated plasma compartment may provide complementary information beyond IFN-γ alone. In addition to IFN-γ, other cytokines and chemokines, including IL-18, IL-10, and CXCL10, have been implicated in HCMV-associated immune responses and may contribute to a broader characterization of antiviral immune competence (54). The potential value of integrating multiple soluble immune mediators is supported by other viral immune monitoring approaches, where combined measurement of IFN-γ and CXCL10 has been used to improve pathogen-specific immune assessment (55, 56).

Notably, these cell populations and markers can be measured in the WB assay. Moreover, the frequencies of “memory-like” NK cells, Vδ1^+^ γδ T cells, and HCMV-specific MBCs remained stable through the end of T-cell stimulation, enabling all parameters to be assessed within a single workflow. Together with the low intra-assay variability observed in our study, these features support the technical robustness and clinical feasibility of the platform.

Compared with established PBMC-based assays, like HCMV-specific IFN-γ ELISpot assays, WB platforms offer several additional practical advantages, including lower blood volume requirements, reduced hands-on processing time, greater suitability for standardization and automation, and preservation of the physiological immune-cell composition, including granulocytes, which can shape interactions with mononuclear cells (6, 57, 58). Compared with our previous protocol (6), omission of BrefA would enable the development of a closed single-tube workflow similar to the QuantiFERON platform, eliminating the need for sterile laboratory infrastructure and allowing for sample handling near the point of care, while facilitating a much broader readout spectrum than commercially available WB-based HCMV-CMI assays. Thereby, the assay combines the optimal sample processing logistics of an exocytosis inhibitor-free WB assay with a multifaceted readout portfolio that previously required isolated PBMCs.

In the future, the assay could be extended to broader research questions, such as integration of transcriptional profiling. We have previously demonstrated nCounter-based gene expression analysis from antigen-stimulated whole blood with α-CD28/α-CD49d co-stimulation (6). We anticipate that a similar approach would also be compatible with the co-stimulation strategy described herein. Furthermore, incorporation of CD45 as an additional flow cytometric marker would facilitate lymphocyte gating and, together with absolute lymphocyte counts from clinical hematology, enable absolute leukocyte quantification, as described previously (59). Moreover, α-CD49d has been shown to improve pre-analytical stability and assay robustness in the presence of immunosuppressive agents (6), and its inclusion in a triple co-stimulatory strategy may further optimize the WB assay. Finally, to avoid prior stimulation and shorten turnaround time, the HLA restriction of multimer-based detection could be addressed by validating panels of the most common HLA-restricted multimers with defined specificity.

Our study has limitations. First, this study did not evaluate the clinical utility of the assay in predicting csCMVi risk. Second, the assay was tested exclusively under optimal conditions, with minimal processing delays, no mechanical stress, and without potential temperature fluctuations during specimen transport. While commercially available WB platforms, such as QuantiFERON, permit transport of stimulated samples for up to 16 hours at room temperature, the feasibility of extended pre-analytic periods with our current protocol remains to be demonstrated. Third, the limited sample size of the validation cohort should be considered as a limitation, as it may restrict the statistical power of the performed analyses. Furthermore, HCMV-specific T-cell responses were assessed using a widely used peptide pool restricted to selected HLA class I alleles. Although this approach covers common HLA alleles differences between individual HLA types and included epitopes may affect detected responses. However, the approach presented in this study focuses on stimulation and incubation of WB near the point of care, thereby avoiding lengthy pre-analytic delay.

Altogether, our versatile, multifaceted, and modular WB immunoassay platform offers substantial potential for translational applications due to its technical simplicity, point-of-care compatibility, and ability to simultaneously assess multiple immune cell subsets and functional endpoints. Thereby, this platform may provide a translationally amenable toolbox to refine post-transplant immune monitoring, improve HCMV risk stratification, help guide decisions on HCMV prophylaxis discontinuation, and support individualized therapeutic decision-making. Beyond HCMV management, the platform is expected to be adaptable to other pathogens (e.g., respiratory viruses or opportunistic molds) and further translational contexts (e.g., studies of post-vaccination immunity), as demonstrated extensively for earlier, primarily T-cell focused iterations of our WB assay (6, 29).

## Data Availability

The raw data supporting the conclusions of this article will be made available by the authors, without undue reservation.
