# Supplementary material for: A multiparameter whole blood platform for assessment of anti-CMV immunity in allogeneic stem cell transplantation recipients
Source: Front Immunol. 2026 Sep 11;17:1872249. doi: 10.3389/fimmu.2026.1872249 (PMC13612563; doi:10.3389/fimmu.2026.1872249)
Supplement: Supplementary file 1 [file SupplementaryFile1.pdf]

Supplementary Methods

1. General note

Unless explicitly stated otherwise, all reagents and antibodies were used according to the manufacturer's instructions. Staining for the PBMC-based methods was performed in the same way as for whole blood, except that only half the amount of antibody specified by the manufacturer was used.

2. Erythrocyte lysis

Human whole blood was collected in 9 mL lithium heparin Monovettes. For erythrocyte lysis, 3 mL of blood was transferred in a 50 mL Falcon tube, followed by addition of 3 mL of EDTA (0.5 M), and incubated for 15 min at room temperature. Next, 6 mL of erythrocyte lysis buffer (Qiagen, Venlo, Netherlands) was added, mixed gently, and centrifuged for 7 min at 600xg at room temperature. The supernatant was removed, then 18 mL of erythrocyte lysis buffer was added and incubated at room temperature for 6 min, inverting occasionally. After further centrifugation at 600xg for 7 min, the supernatant was removed. Next, 6 mL of PBS was added and centrifuged for 5 min at 400xg at room temperature, and again the supernatant was discarded. The cells were resuspended in 3 mL of RPMI 1640 medium with 5% human serum and counted.

The cells were divided for subsequent staining: 5×10^6 cells for B cell analyses, 5×10^5 cells each for positive and negative MACSimer approaches, and 3×10^5 cells for γδ T and NK cells.

3. Testing of different activation markers

For evaluation of additional activation markers during AIM stimulation in our whole-blood AIM assay without BrefA and α-CD28/α-CD40 co-stimulation, cells were processed analogously to the AIM staining procedure described in the main manuscript. Following overnight stimulation with HCMV peptide pools and an unstimulated negative control, erythrocyte lysis was performed as described above. Cells were stained using the same AIM staining procedure, with additional surface staining for CD25, HLA-DR, and CD134 (**Table S1**). CD137 and CD154 were stained both extracellularly and intracellularly as described for the AIM assay.

4. γδ T and NK cells

The cells for the γδ T and NK cells were stained with the related antibodies (Table S1) for 30 min at 4 °C after erythrocyte lysis. The cells were then washed twice in wash buffer (D-PBS + 1% FCS) and measured in 100 µL wash buffer on the flow cytometer.

5. HLA-reactive multimers

5.1 Coupling multimers to the matching HLA peptide

For the analysis of HCMV-reactive $CD8^+$ T cells, the HLA-specific MACSimers were coupled with the HLA-reactive peptide the day before. The following kits were used for this purpose: MHC MACSimer A01:01 Flex Kit, human PE; MHC MACSimer A02:01 Flex Kit, human PE; MHC MACSimer B07:02 Flex Kit, human PE (all from Miltenyi Biotec, B.V. & Co. KG, Bergisch Gladbach, Germany), and

mixed with the respective HLA-matched peptide: CMVUL44 (VTEHDTLLY) HLA*A01:01; CMVpp65 (NLVPMVATV) HLA*A02:01; and CMVpp65 (RPHERNGFTVL) HLA*B07:02 (all from Miltenyi Biotec). The reagents were brought to room temperature, and 15 µL of MACSimerFlex reagent with 0.5 µL peptide (10 mmol/L) or negative control was added to 0.5 mL Eppendorf tubes per batch, 9.5 µL loading buffer was added and carefully pipetted up and down (do not vortex). Incubation was performed for 16–18 h at room temperature in the dark.

The tubes were then centrifuged at 3300xg for 5 min at room temperature, and the supernatant was stored at 4 °C protected from light.

5.2 Staining of HLA-reactive multimers

For each MACSimer approach, 5×10^5 cells were incubated with 2.5 µL of the coupled MACSimers for 10 min at room temperature in the dark and then washed twice with wash buffer (D-PBS + 0.5% BSA + 5 mM 0.5M EDTA) (5 min, 300 xg, 4 °C). The cells were then incubated with the remaining antibodies (**Table S1**). After incubation, the cells were washed twice again and measured in 100 µL wash buffer on the flow cytometer.

6. HCMV-specific memory B cells

6.1 Biotinylation of HCMV-specific glycoproteins

HCMV glycoproteins were used as probes to identify reactive memory B cell populations. The HCMV trimer (gH/gL/gO complex, The Native Antigen Company, Kidlington, UK), the pentamer (gH, gL, UL128, UL130, UL131A complex, The Native Antigen Company) and glycoprotein B (gB, SinoBiological, Beijing, China) were biotinylated with EZ-Link Sulfo-NHS-Biotin (Thermo Scientific, Waltham, Massachusetts). Incubation was performed for 30 min at room temperature with a 20-fold molar ratio of the biotin reagent, after which the proteins were desalted using Zeba Spin Columns (Thermo Scientific). The biotinylated proteins were enriched with 15% glycerol (final concentration) and stored at 4°C. Before use, streptavidin PE and APC were bound to the probes.

6.2 Coupling of biotinylated HCMV-specific glycoproteins to streptavidin

The cells were then supplemented with the previously coupled SA-conjugated antigens gB-SA PE, Tri-SA PE, Pent-SA PE, Streptavidin BV510 (diluted 1:100), gB-SA APC, Tri-SA APC, and Pent-SA APC, together with 50 µL Brilliant Stain Buffer (Becton, Dickinson and Company, Franklin Lakes, NJ, USA) and D-biotin (diluted 1:100; 10 mM; ThermoFisher). After 60 min incubation at 4 °C, the cells were washed twice.

6.3 Staining of HCMV-specific MBCs

The cells for the B-cell approach were adjusted to 5×10^6 cells and incubated with 100 µL staining buffer (D-PBS + 2% FCS + 0.4% 0.5M EDTA), 5 µL FcR blocking reagent (Miltenyi Biotec), and L/D eFluor780 for 30 min at 4°C followed by two washing steps with staining buffer. The cells were then supplemented with the previously coupled SA-conjugated antigens gB-SA PE, Tri-SA PE, Pent-SA PE, Streptavidin BV510 (diluted 1:100), gB-SA APC, Tri-SA APC, and Pent-SA APC, together with 50 µL Brilliant Stain Buffer (Becton, Dickinson and Company, Franklin Lakes, NJ, USA) and D-biotin (diluted 1:100; 10 mM; ThermoFisher). After 60 min incubation at 4 °C, the cells were washed twice. This was followed by final staining with Brilliant Stain Buffer (50 µL), Staining Buffer (50 µL) and the remaining

antibodies (**Table S1**). Incubation was performed for 30 min at 4 °C, after two washing steps cells were measured in 200 µL staining buffer on a flow cytometer.

7. Day two quantification of γδ T cells, NK cells and HCMV-specific B cells

To test whether γδ T and NK cells and HCMV-specific B cells could be stained and measured on day two in parallel to the AIM assay, pre-prepared stimulation tubes containing only RPMI 1640 medium were prepared. For γδ T and NK cells, 50 µL of medium was transferred into the Monovettes and stored at −20 °C as described above for stimulation. Subsequently, 0.45 mL of whole blood was injected into each tube. For HCMV-specific B cell analysis, 200 µL of medium was transferred and stored in Monovettes, followed by injection of 1.8 mL of whole blood. The tubes were incubated overnight at 37 °C, analogous to the AIM assay. On the next day, erythrocyte lysis was performed as described above, with reagent volumes adjusted for the respective blood volumes. Staining was performed analogously to the procedures described above.

8. Evaluation of intraassay precision

For assessment of intraassay precision, blood from four HCMV-seropositive individuals was processed in duplicate, stained, and measured in parallel on day two.

7. Antigen specific T cell enrichment

For antigen specific T cell enrichment (ARTE) of HCMV-reactive CTLs or Th cells, four times the amount of whole blood was stimulated overnight and erythrocyte lysis was then performed.

To label the activated T cells, CD137 PE and biotinylated CD154 (Miltenyi) were added and incubated for 15 min at 4°C. The cells were then washed once, and those not intended for ARTE were resuspended in 1 mL staining buffer and stored at 4°C.

For ARTE enrichment, the cell pellets were resuspended in 80 µL ice-cold MACS buffer (D-PBS + 0.5% FCS + 2 mM EDTA) and 20 µL anti-biotin beads and anti-PE microbeads were added. The mixture was incubated for 15 min at 4°C in the refrigerator. The cells were then washed with 1 mL of ice-cold MACS buffer, the supernatant was removed, and the cells were resuspended in 500 µL of MACS buffer.

Magnetic cell separation was performed using LS columns (Miltenyi), which were pre-rinsed with 3 mL of ice-cold MACS buffer. The labeled cells were applied, the column was rinsed three times with 3 mL of MACS buffer each time, and the flow was discarded. The column was then removed from the magnet and the cells were eluted with 1 mL MACS buffer.

After ARTE, all cells (with and without ARTE) were centrifuged at 300×g, 5 min, 4 °C. The pellet was stained with the remaining antibodies (**Table S1**) and 2 µL anti-biotin APC (Miltenyi) for 30 min at 4 °C. The cells were then washed once, and fixation and intracellular staining were performed in the same way as for the AIMs.

Supplementary Tables

Table S1. Antibodies and manufacturers used for flow cytometric analysis of different immune cell populations. The panel includes markers for γδ T cells, NK cells, AIMs, ARTEs, MHC multimers, and HCMV-specific MBCs.

Fluorochromes	Multimers	AIMs	B cells	ARTE	NK/gd T cells	Activation-markers
**FITC**	X	X	IgG[5]	CD45[1]	TCRVδ1[1]	HLA-DR[1]
**PE**	Multimers[1]	CD137[1]	Streptavidin[1]	CD137[1]	TCRγ/δ[1]	CD137[1]
**ECD**	X	X	CD27[5]	CD45RA[4]	X	CD134[1]
**PerCP-Vio770**	CD14/CD20[1]	X	X	X	X	CD25[3]
**PE-Cy7**	X	X	IgA[1]	PD-1[3]	CD159c[1]	X
**APC**	X	CD154[1]	Streptavidin[1]	CD154[1]	CD57[1]	CD154[1]
**VioB R720**	CD8[1]	CD8[1]	CD21[5]	CD8[1]	CD3[3]	CD8[1]
**eFluor ™ 780**	L/D[2]	L/D[2]	L/D[2]	L/D[2]	L/D[2]	L/D[2]
**VioBlue**	X	X	IgM[5]	CD69[1]	X	CD69[1]
**VioGreen**	CD3[1]	CD3[1]	Streptavidin[1]	CD3[1]	CD56[5]	CD3[1]
**VioB V600**	CD4[1]	CD4[1]	IgD[5]	CD4[1]	X	CD4[1]
**BV650**	X	X	CD20[5]	CCR7[5]	X	X

Manufacturers: 1 = MACSimers, Miltenyi Biotec, Bergisch Gladbach, Germany; 2 = Invitrogen, Carlsbad, CA, USA; 3 = Becton, Dickinson and Company (BD), Franklin Lakes, NJ, USA; 4 = Beckman Coulter Biomedical, Brea, CA, USA; 5 = BioLegend Inc., San Diego, CA, USA

Table S2. AIM assay comparison of background frequencies between HCMV-seropositive and HCMV-seronegative patients.

Median frequencies of $CD137^+$ and $CD154^+$ activated T cells are shown for unstimulated controls from HCMV-seronegative and HCMV-seropositive patients, as well as for HCMV peptide pool-stimulated HCMV-seronegative patients. Whole blood (WB)- and PBMC-based measurements, different costimulation conditions (α-CD28/α-CD40 vs. α-CD28/α-CD49d), and BrefA conditions are compared. p values were calculated using the Wilcoxon matched-pairs signed-rank test. For the comparison of BrefA-containing and BrefA-free conditions, N = 3 HCMV-seronegative and N = 6 HCMV-seropositive individuals were analyzed. For the comparison of WB- and PBMC-based measurements, N = 12 HCMV-seropositive patients were included.

	HCMV-seropositive patients		HCMV-seronegative patients	
**Condition**	**Median unstimulated**	**P**	**Median unstimulated**	**Median HCMV-peptide pool**
CD137+ αCD28/αCD40 Bref-	0.07	0.063	0	0.15
CD137+ αCD28/αCD49d Bref+	0.225		0.1	0
CD154+ αCD28/αCD40 Bref-	0.01	0.438	0	0
CD154+ αCD28/αCD49d Bref+	0.02		0	0
CD137+ WB	0.065	0.5		
CD137+ PBMCs	0.065			
CD154+ WB	0.04	0.12		
CD154+ PBMCs	0.015			

Supplementary Figures

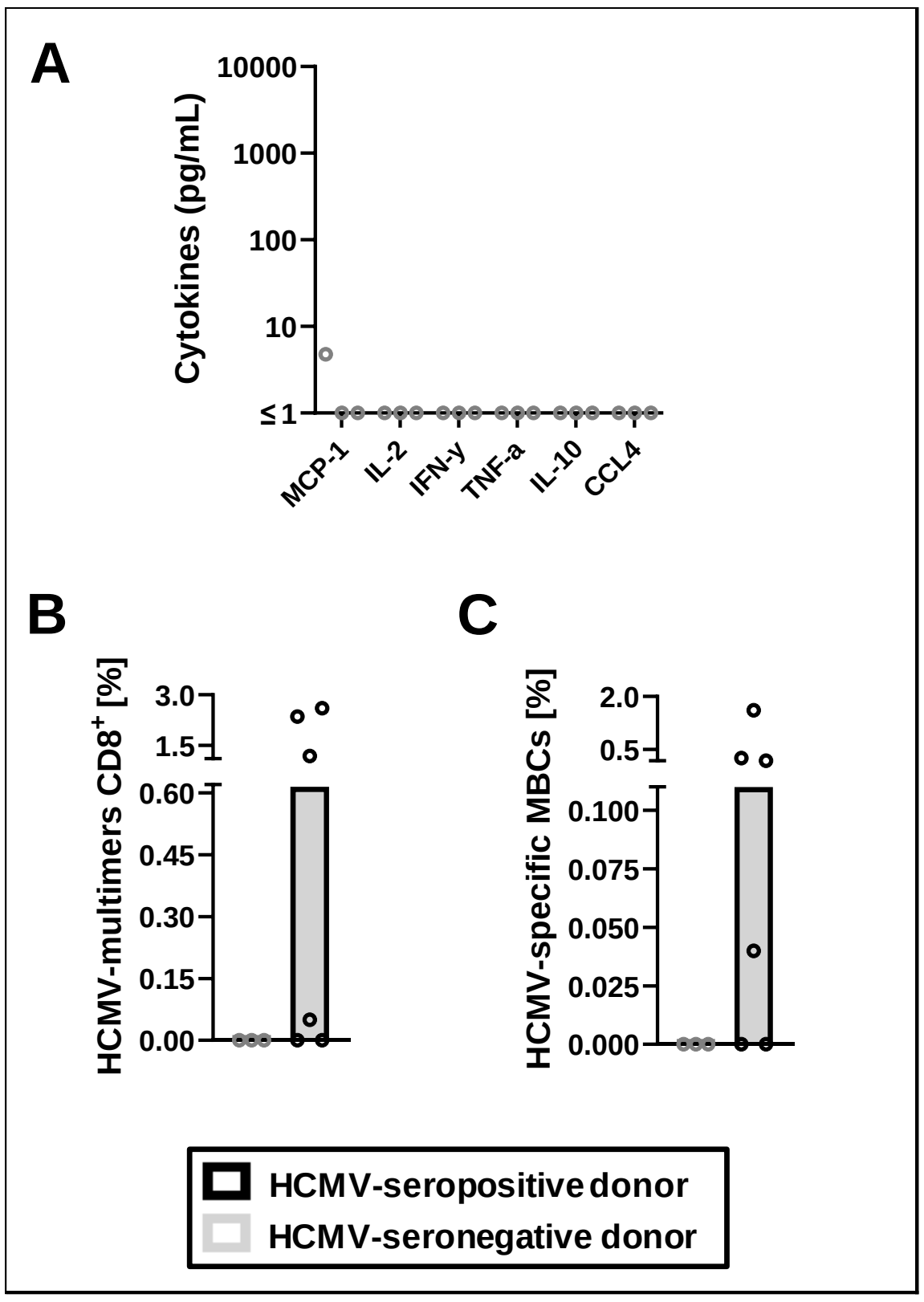

Figure S1. The whole blood assay shows high specificity between HCMV-seropositive and HCMV-seronegative samples.

(**A**) Concentration of cytokines after multiplex of MCP-1, IL-2, IFN-γ, TNF-α, IL-10 and CCL4 after stimulation with HCMV-Peptide Pool for seronegative (grey) individuals. **(B-C**) Comparison of HCMV-seronegative and HCMV-seropositive (black) donors, showing frequencies of HCMV-reactive cytotoxic T lymphocytes (CTLs; $CD137^{+}$ among $CD8^{+}$, **B**) detected by HCMV-reactive multimers and HCMV-specific memory B cells (MBCs, **C**). (**A-C**) N = 3 HCMV-seronegative (**B-C**) and N = 6 HCMV-seropositive recipients analyzed between days 129 and 665 after alloSCT.

<u>Abbreviations:</u> alloSCT = allogeneic stem cell transplantation; CTLs = cytotoxic T lymphocytes; HCMV = human cytomegalovirus; IFN-γ = interferon gamma; IL = interleukin; MCP-1 = monocyte chemoattractant protein-1; MBCs = memory B cells; PBMCs = peripheral blood mononuclear cells; TNF-α = tumor necrosis factor alpha; WB = whole blood.

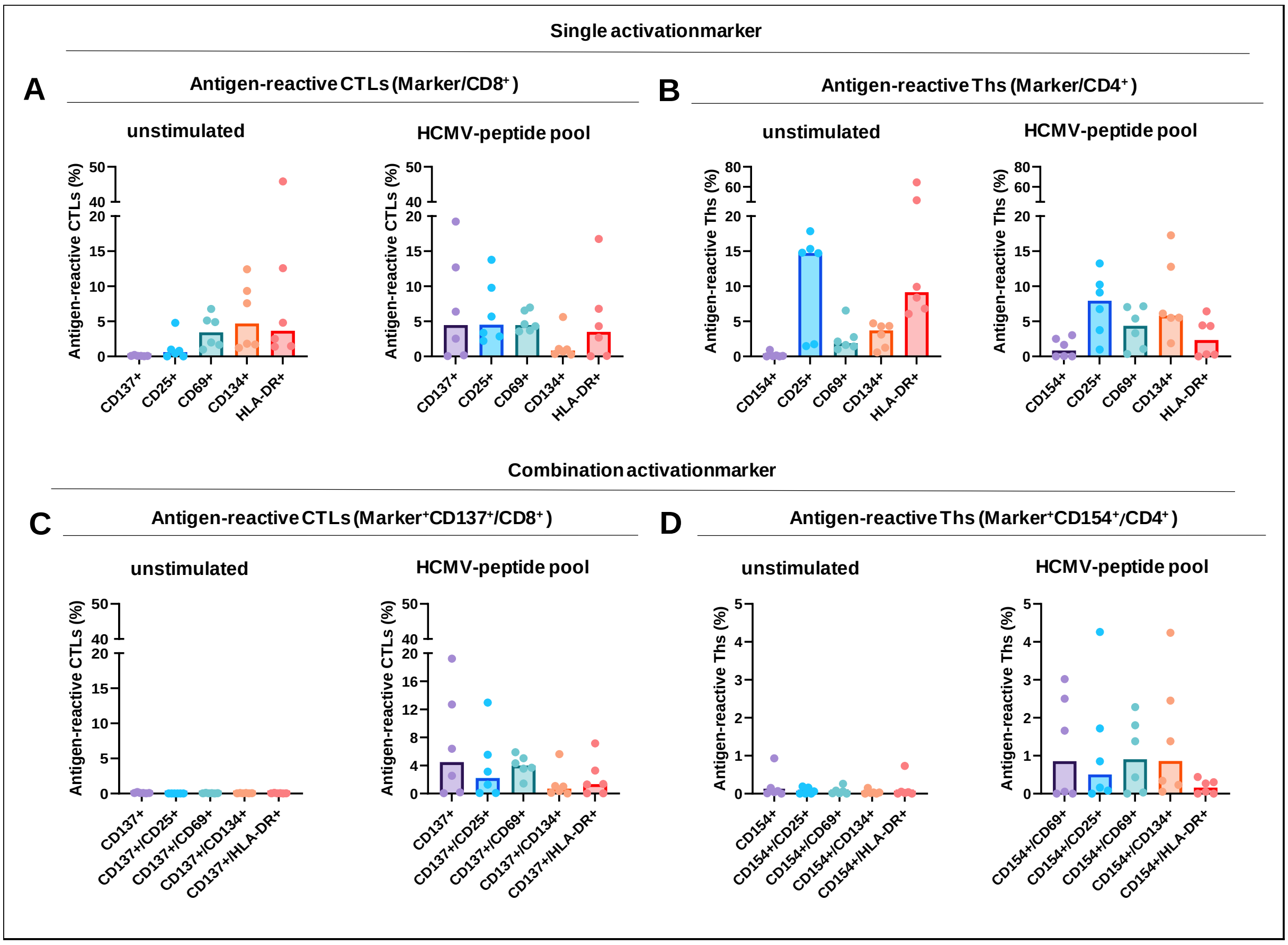

Figure S2. Multiple activation markers and combinatorial activation marker strategies are detectable in the whole-blood assay following HCMV peptide stimulation.

(**A-B**) Frequencies of CD137^{+} (CTLs), CD154^{+} (Ths), CD25^{+}, CD69^{+}, CD134^{+}, and HLA-DR^{+} cells in unstimulated control samples (left) and following HCMV-peptide pool stimulation (right) for cytotoxic T lymphocytes (CTLs, **A**) and T helper cells (Ths, **B**). (**C-D**) Frequencies of CTLs co-expressing CD137 with CD25, CD69, CD134, or HLA-DR (**C**) and Ths co-expressing CD154 with CD25, CD69, CD134, or HLA-DR (**D**) in unstimulated control samples (left) and following HCMV peptide pool stimulation (right). (**A-D**) N = 6 HCMV-seropositive recipients analyzed between day 102 and year 3 after alloSCT.

Abbreviations: alloSCT = allogeneic stem cell transplantation; CTLs = cytotoxic T lymphocytes; HCMV = human cytomegalovirus; Ths = T helper cells; WB = whole blood.

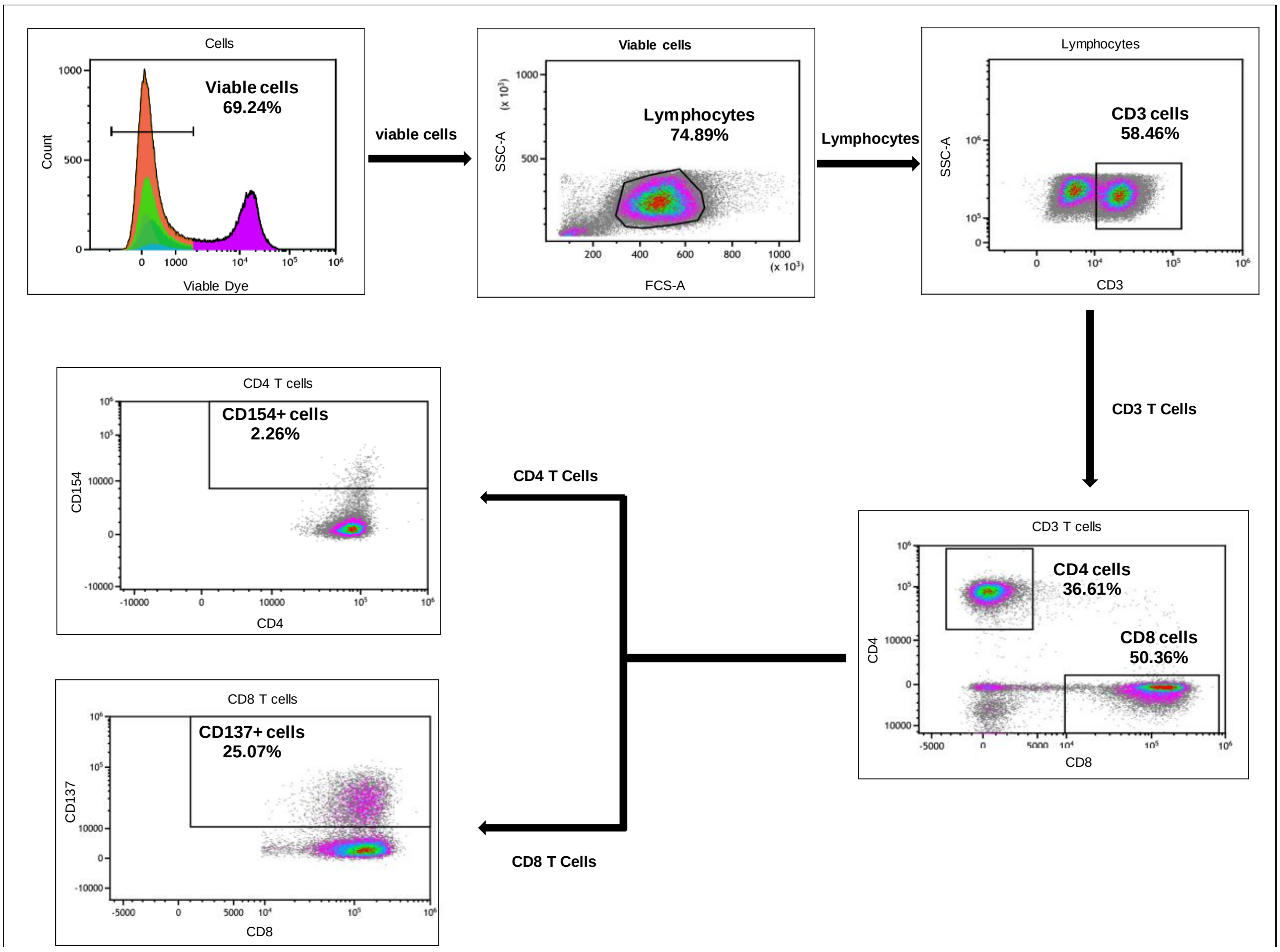

Figure S3. Gating strategy for the identification of antigen specific CD4⁺- and CD8⁺-T cells in whole blood

Abbreviations: CD = cluster of differentiation; FSC = forward scatter; SSC = side scatter.

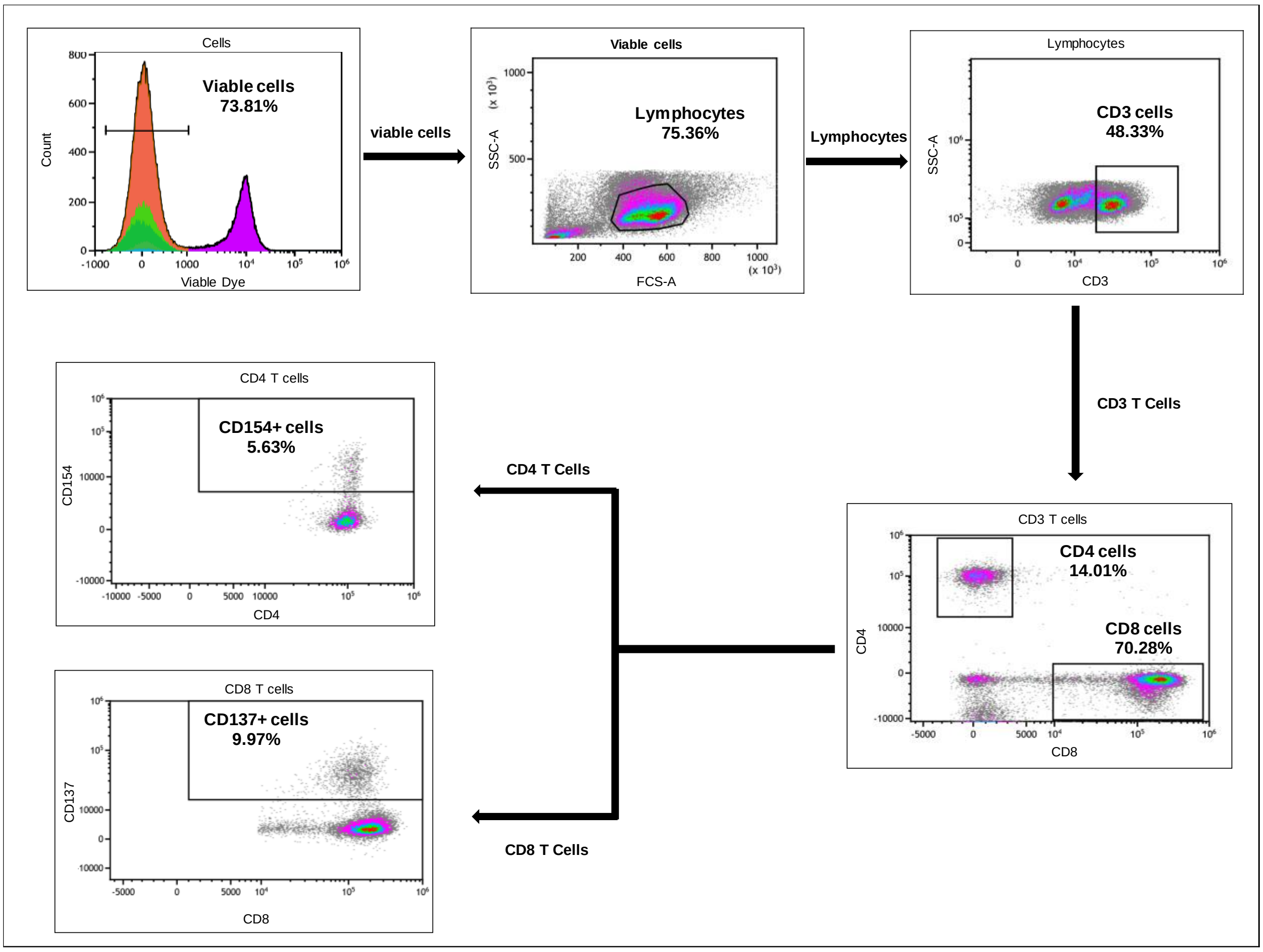

Figure S4. Gating strategy for the identification of antigen specific CD4^{+}- and CD8^{+}-T cells in PBMCs

Abbreviations: CD = cluster of differentiation; FSC = forward scatter; SSC = side scatter.

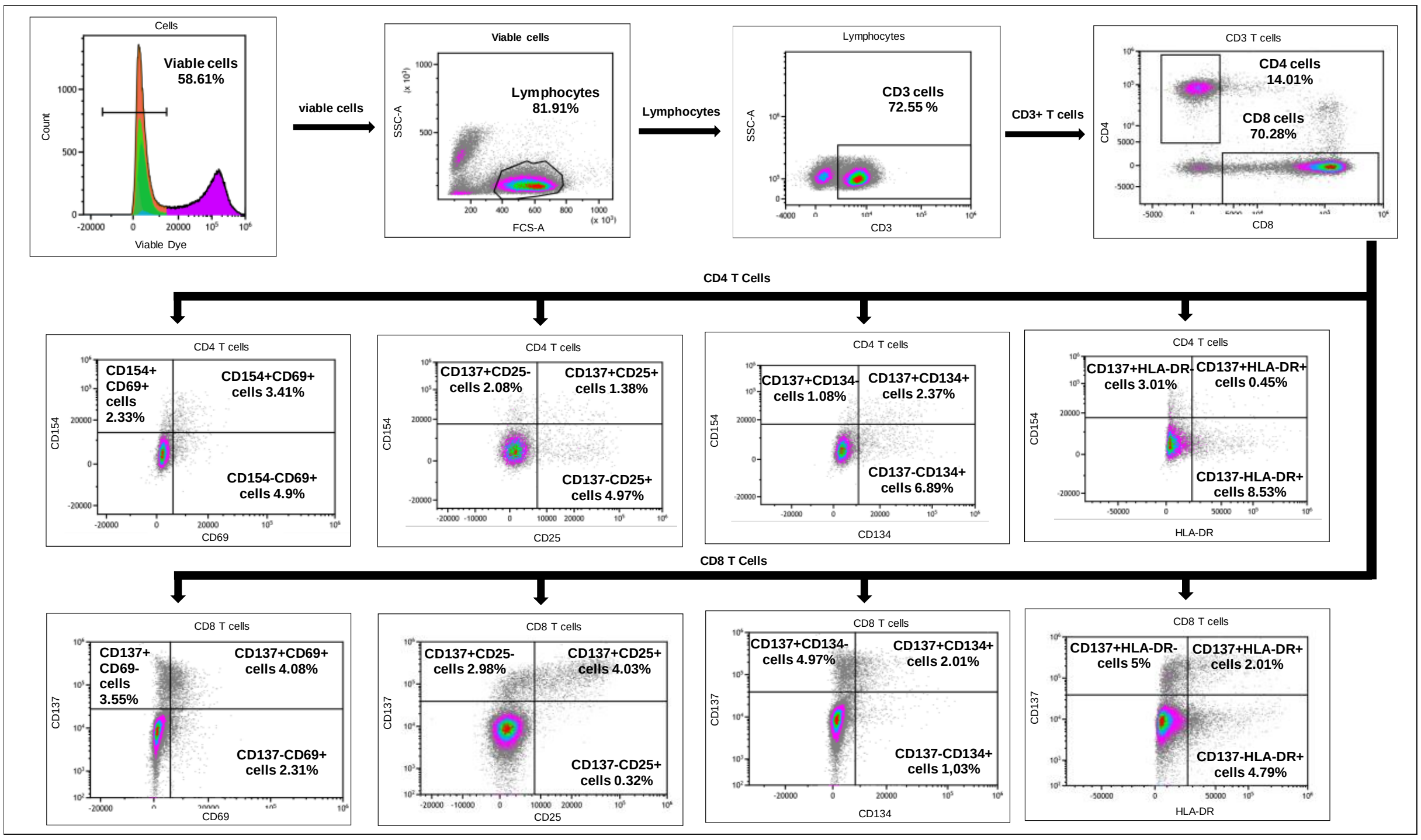

Figure S5. Gating strategy for the evaluation of different activationmarkers in whole blood

Abbreviations: CD = cluster of differentiation; FSC = forward scatter; HLA-DR = Human leukocyte antigen-DR; SSC = side scatter

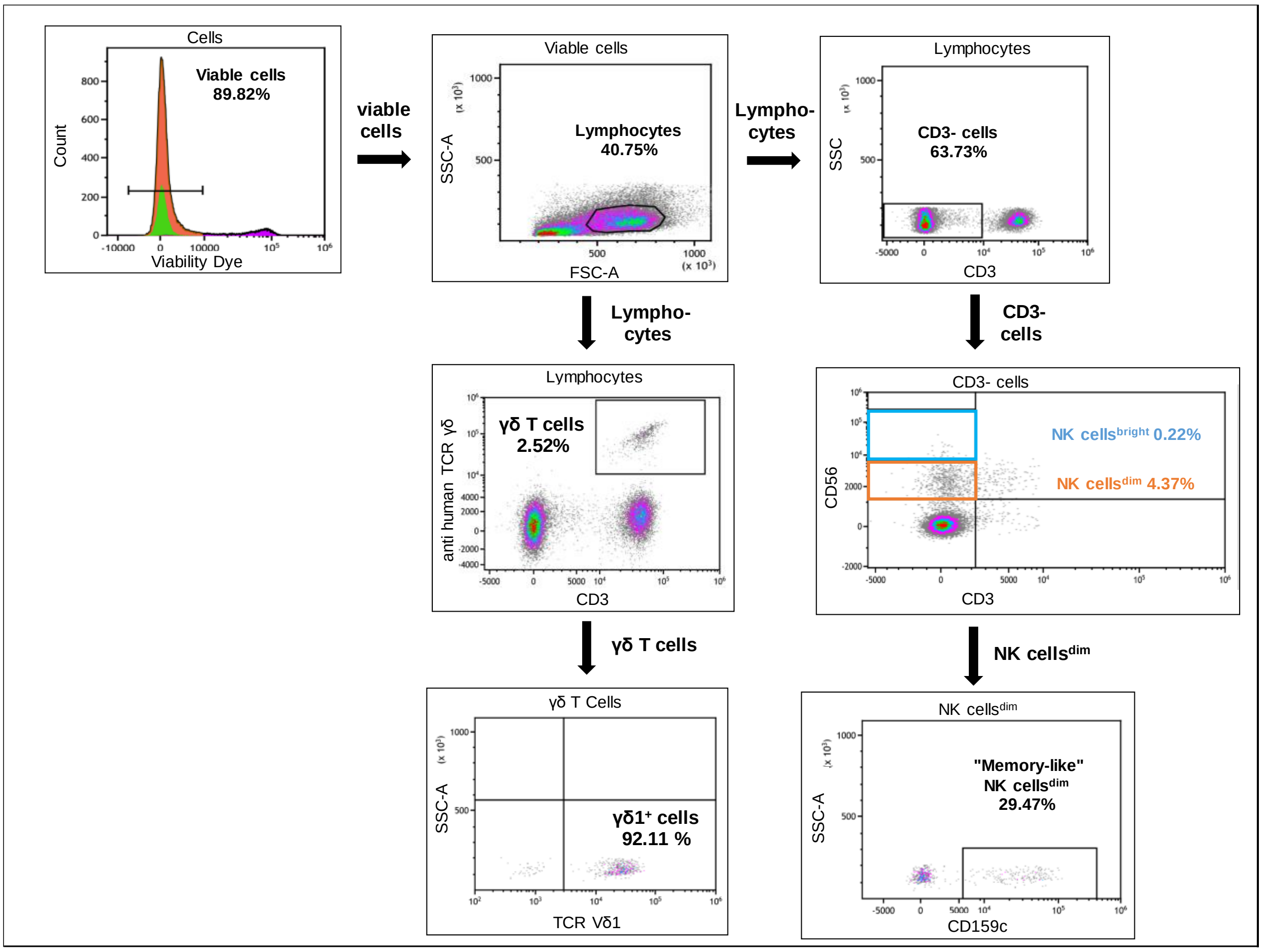

Figure S6. Gating strategy for the identification of γδ T cells and ("memory-like") NK cells in whole blood

Abbreviations: CD = cluster of differentiation; FSC = forward scatter; TCR = T-cell receptor; SSC = side scatter.

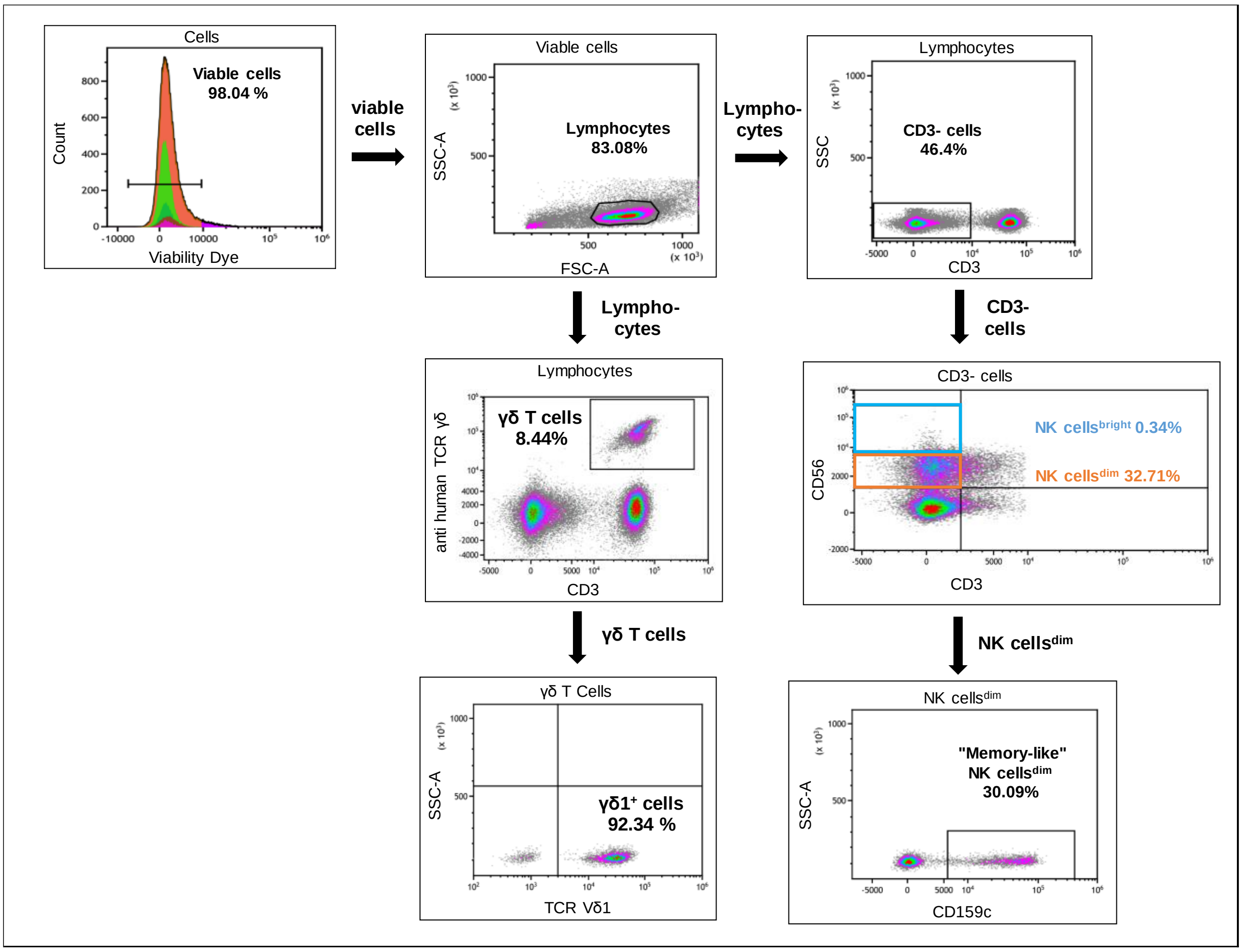

Figure S7. Gating strategy for the identification of γδ T cells and ("memory-like") NK cells in PBMCs

Abbreviations: CD = cluster of differentiation; FSC = forward scatter; TCR = T-cell receptor; SSC = side scatter.

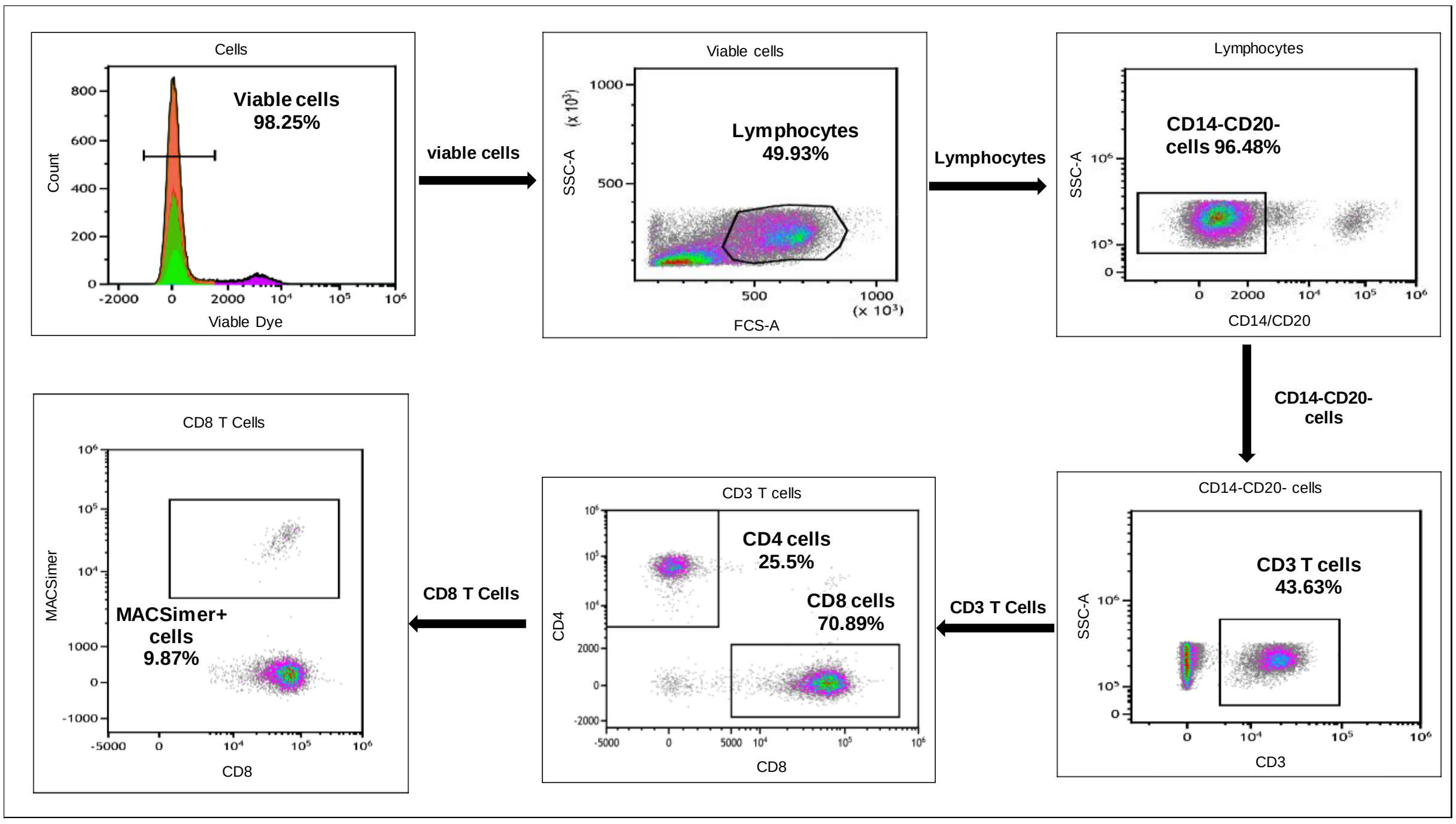

Figure S8. Gating strategy for the identification of HCMV-specific CD8⁺ T cells using HLA class I multimers in whole blood

HCMV-specific $CD8^+$ T cells were identified using HLA-matched multimers with the corresponding peptides. HLA*A01:01: CMVUL44 (VTEHDTLLY); HLA*A02:01: CMVpp65 (NLVPMVATV); HLA*B07:02: CMVpp65 (RPHERNGFTVL)

Abbreviations: CD = cluster of differentiation; FSC = forward scatter; MACSimer = HLA class I multimers; SSC = side scatter.

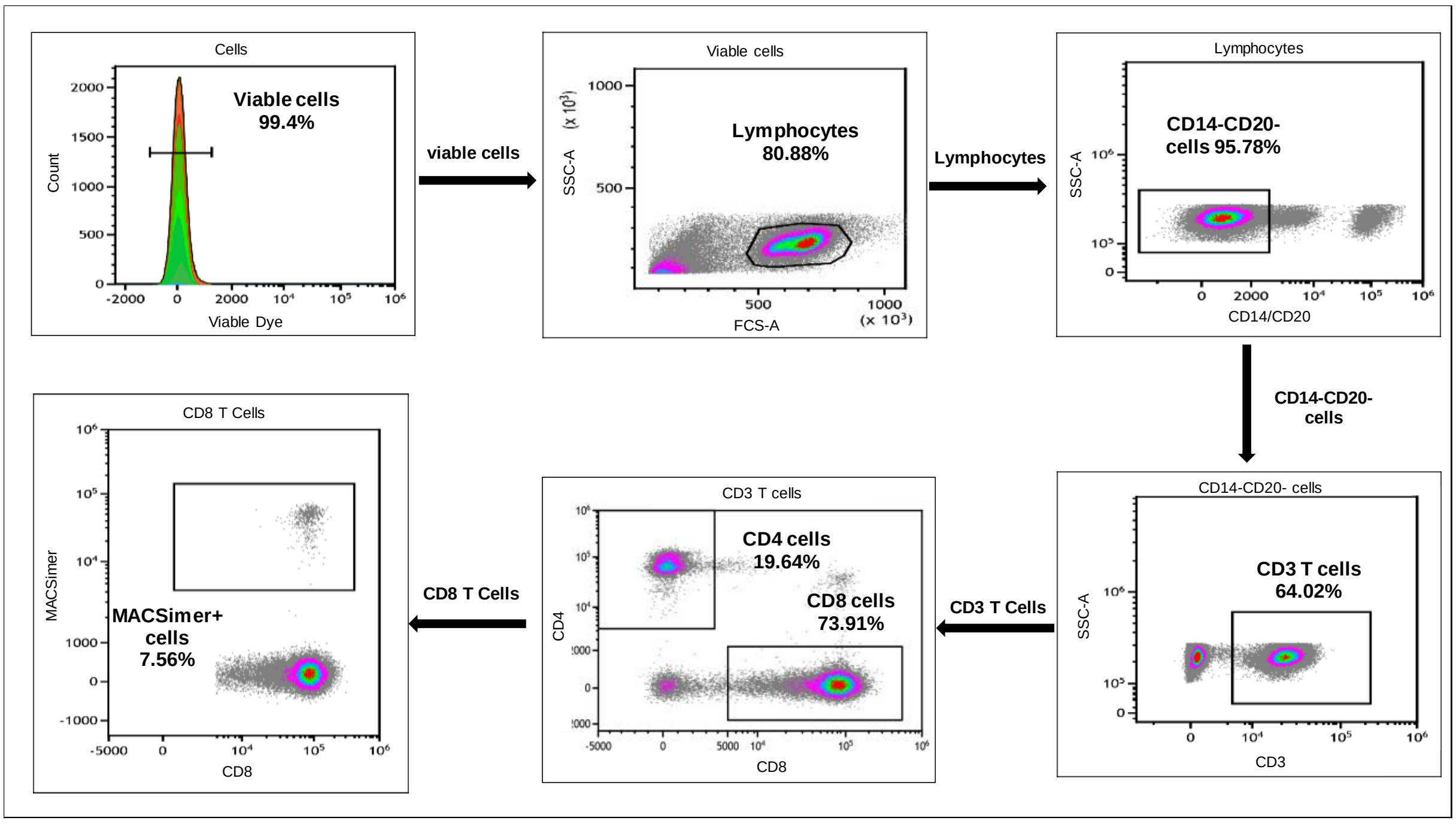

Figure S9. Gating strategy for the identification of HCMV-specific CD8⁺ T cells using HLA class I multimers in PBMCs

HCMV-specific CD8⁺ T cells were identified using HLA-matched multimers with the corresponding peptides. HLA*A01:01: CMVUL44 (VTEHDTLLY); HLA*A02:01: CMVpp65 (NLVPMVATV); HLA*B07:02: CMVpp65 (RPHERNGFTVL)

Abbreviations: CD = cluster of differentiation; FSC = forward scatter; MACSimer = HLA class I multimers; SSC = side scatter.

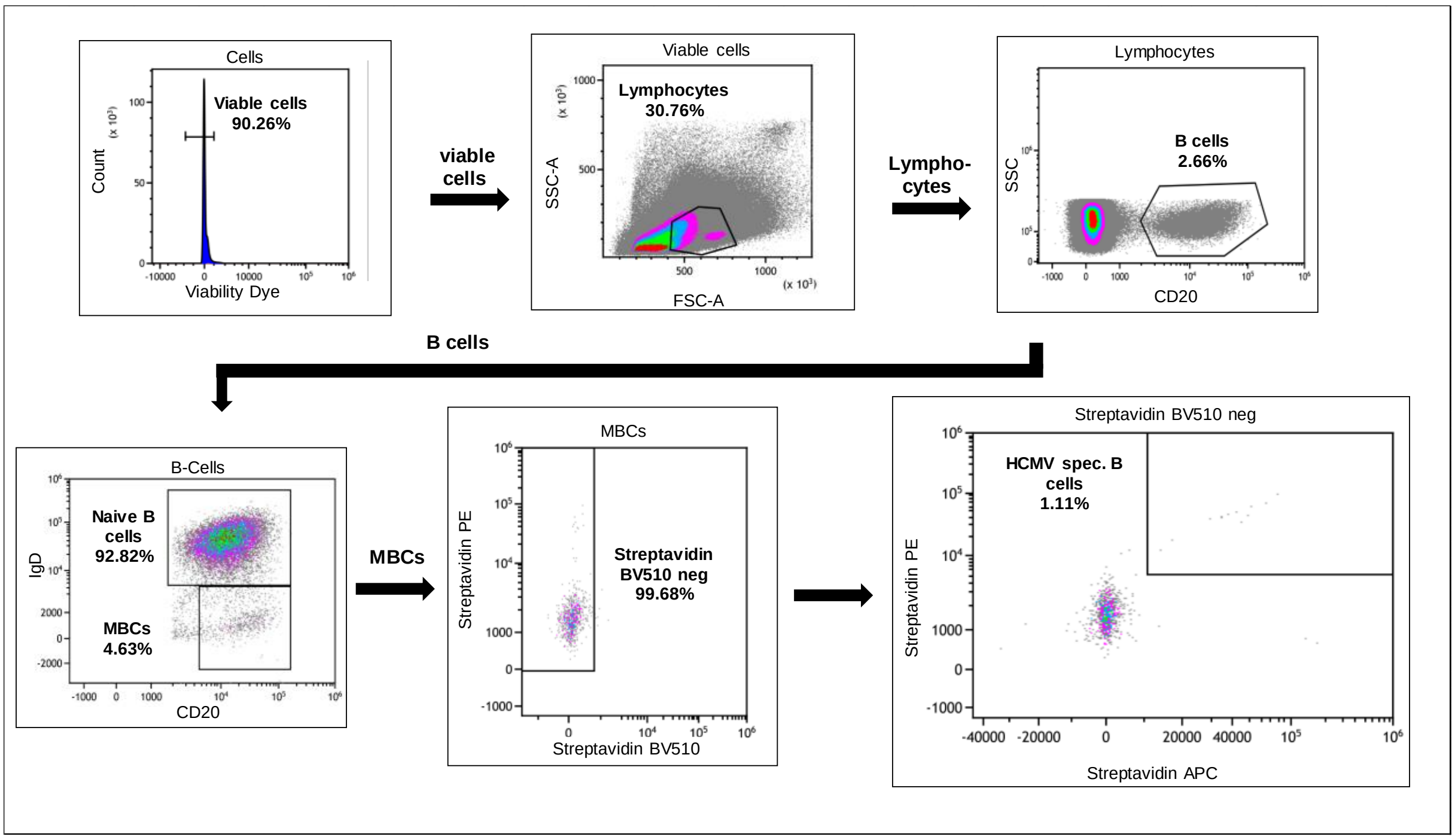

Figure S10. Gating strategy for the identification of HCMV-specific MBCs in whole blood

Abbreviations: CD = cluster of differentiation; FSC = forward scatter; HCMV = human cytomegalovirus; MBCs = memory B cells; HCMV-spec. = HCMV-specific; IgD = immunoglobulin D; SSC = side scatter.

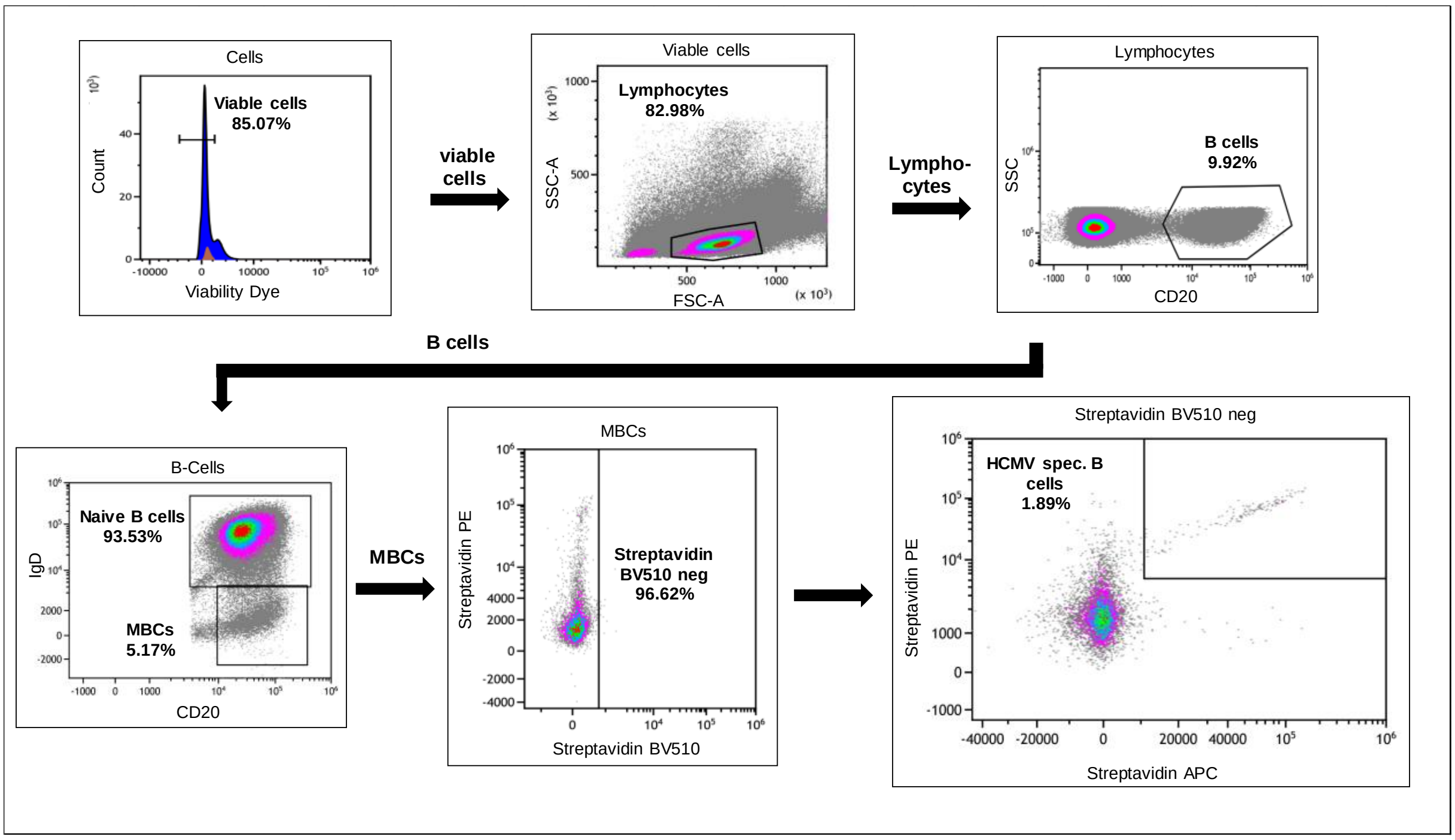

Figure S11. Gating strategy for the identification of HCMV-specific MBCs cells in PBMCs

Abbreviations: CD = cluster of differentiation; FSC = forward scatter; HCMV = human cytomegalovirus; MBCs = memory B cells; HCMV-spec. = HCMV-specific; IgD = immunoglobulin D; SSC = side scatter.

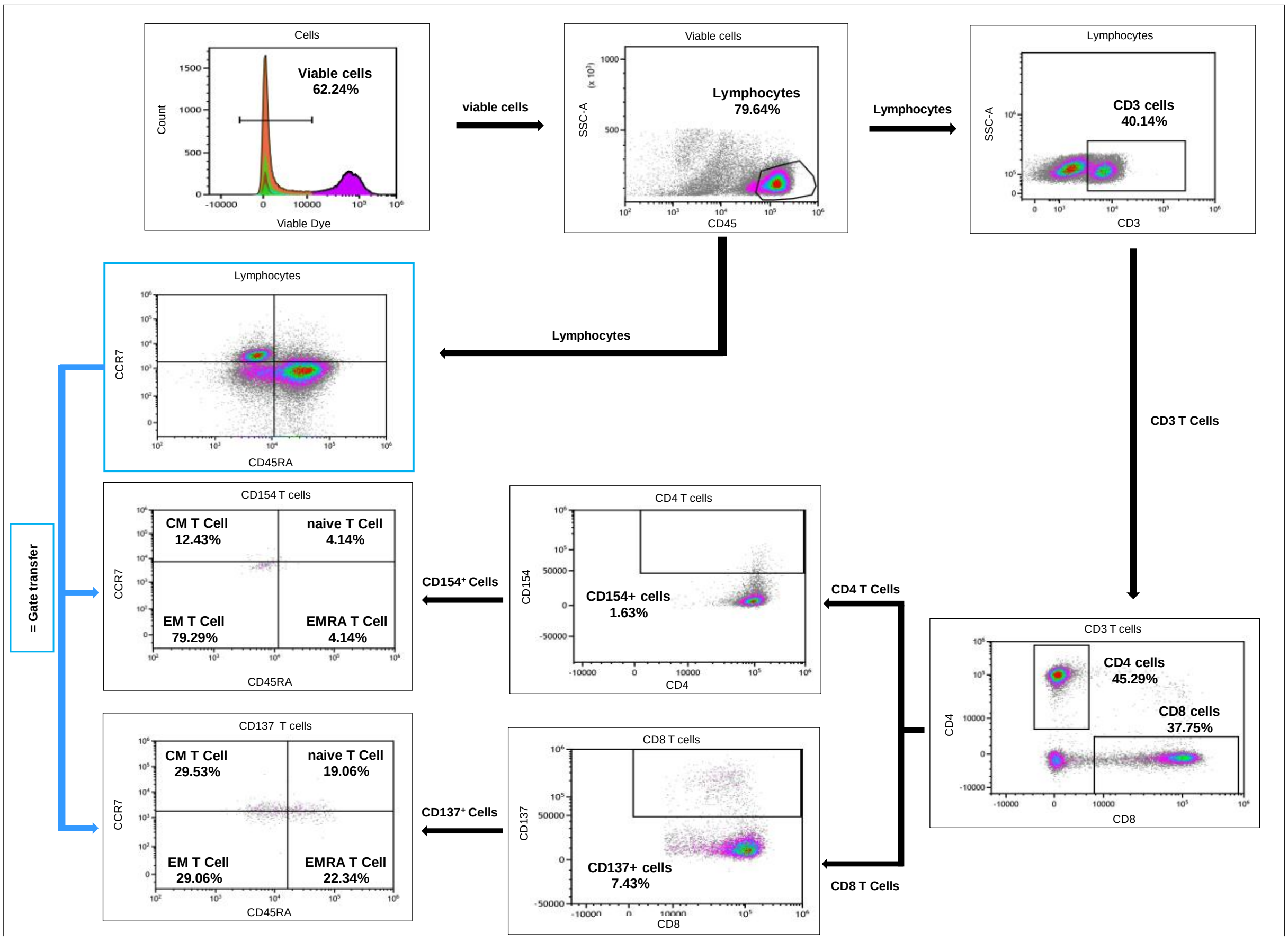

Figure S12. Gating strategy for the identification of antigen specific CD4+- and CD8+-T cells as well as their memory status in whole blood

Abbreviations: CD = cluster of differentiation; FSC = forward scatter; SSC = side scatter.
